# Tailoring Bayesian Additive Regression Trees (BART) for environmental mixture studies

**DOI:** 10.1371/journal.pone.0348002

**Published:** 2026-05-11

**Authors:** Kaizong Ye, Zhen Chen, Shanshan Zhao

**Affiliations:** 1 Biostatistics and Computational Biology Branch, National Institute of Environmental Health Sciences, National Institutes of Health, Durham, North Carolina, United States of America; 2 Biostatistics and Bioinformatics Branch, Division of Population Health Research, Division of Intramural Research, Eunice Kennedy Shriver National Institute of Child Health and Human Development, National Institutes of Health, Rockville, Maryland, United States of America; Sichuan University, CHINA

## Abstract

**Background:**

Various methods have been developed to investigate the complex and collective effects of environmental mixtures on human health. Tree ensemble methods, such as Bayesian Additive Regression Trees (BART), are known for their stability and accuracy in variable selection and outcome prediction for high-dimensional correlated data in the statistical literature, but their use has not been well studied for environmental mixtures.

**Methods:**

We tailored the original BART model for environmental mixtures analysis to achieve both robust identification of toxic agents and accurate prediction of health outcomes. Our modified BART approach allowed for a smooth response surface and incorporated covariate adjustment for both continuous and binary outcomes. It supported both component-wise variable selection and hierarchical variable selection to accommodate scientifically meaningful groupings of chemicals. To facilitate interpretation, we used a Generalized Additive Model (GAM) approximation to quantify the marginal contributions of individual chemicals. The performance of the modified BART was evaluated through simulations and a case study with the National Health and Nutrition Examination Survey (NHANES) 2001–2002 data to examine the effects of persistent organic pollutants (POPs) on leukocyte telomere length. All results were compared with the Bayesian Kernel Machine Regression (BKMR), a widely used method in mixtures analysis.

**Results:**

Our simulation studies demonstrated that the modified BART produced results comparable to or superior to BKMR in recovering the true exposure-response surface for both continuous and binary outcomes, with $R^{\text{2}}$ consistently above 0.7. Specifically, when chemical groups were considered, modified BART with hierarchical variable selection achieved higher $R^{\text{2}}$ (0.82–0.99 for continuous outcomes and 0.73–0.95 for binary outcomes) than BKMR (0.59–0.67 and 0.47–0.59, respectively), on independent test datasets. Modified BART also reduced the computational time by 70% to 99.8% compared to BKMR. Both methods effectively identified relevant chemical groups under hierarchical variable selection, but modified BART more effectively distinguished important components within groups. In the NHANES case study, three chemicals, including 2,3,4,7,8-pncdf, PCB126 and PCB169, were identified by modified BART as having near-linear positive effects on leukocyte telomere length based on GAM approximation plots.

**Conclusions:**

Modified BART is a robust and scalable response surface model alternative to BKMR for analyzing environmental mixtures data. It is particularly advantageous for large datasets, binary outcomes, and grouped chemicals. GAM approximation provides practical insights into interpreting individual chemical effect estimated from complex response surface models.

## Introduction

Humans are simultaneously exposed to a wide range of environmental chemicals through air, water, food, consumer products, and other pathways. Studying mixtures of these environmental exposures is crucial for advancing our understanding of both their individual and combined impacts on human health [1–3]. Key objectives of environmental mixtures studies include characterizing exposure patterns, identifying toxic agents within mixtures, and quantifying their individual and cumulative effects. It is also desirable to incorporate meaningful chemical grouping information into analysis, since chemicals within the same group often co-exist, share biological pathways, and respond similarly to interventions. However, environmental mixtures data are often of high-dimensional, highly correlated, and exhibit complex interactions and non-linear relationships with health outcomes. These features present major challenges for traditional regression methods. Therefore, it is important to develop specialized statistical methods to gain deeper insights into the health effects of environmental mixtures.

Various regression models have been used to explore the association between environmental mixtures and health outcomes, including regularized regressions, index models, and response surface models. Regularized regressions, such as elastic net regression [4] and LASSO (least absolute shrinkage and selection operator) [5], can be used to identify relevant chemicals through variable selection. Index models, such as weighted quantile sum regression (WQS) [6], quantile-based g-computation (qgcomp) [7], and partial linear single index model (PLSI) [8], use weighted indices of chemicals to explore their cumulative effects on health outcomes. Response surface models are a class of flexible models designed to explore the non-linear relationships between exposures and various outcomes in different research areas [9–16]. In particular, Bayesian Kernel Machine Regression (BKMR) [17,18] is one of the most popular approaches for mixtures analysis, primarily due to its model flexibility and visualization tools. It has been used in diverse contexts to investigate the health effects of air pollution [19,20], heavy metals [21], and endocrine disruptors [22], among others. However, kernel methods are generally computationally intensive, and BKMR can have convergence issues due to its reliance of Markov Chain Monte Carlo (MCMC), which can become particularly burdensome for high-dimensional or large datasets.

There is growing interest in the use of tree-based models in both statistical and biomedical researches [23–27], primarily due to their ability to recursively partition the predictor space [28] to improve estimation precision and capture complex non-linear relationships. However, individual trees can be unstable, motivating the development of ensemble methods that integrate multiple weak learners. Among these, Bayesian Additive Regression Trees (BART) model [29] uses MCMC sampling to approximate the posterior distribution over trees. Its variant, soft BART, extends BART by incorporating probabilistic splits and sparsity-inducing priors to generate smooth response surfaces for high-dimensional data [30,31]. These features make soft BART particularly suitable for mixtures analysis, where chemical effects tend to vary gradually rather than abruptly, and a small subset of exposures contributes most to the overall effect. BART also offers computational efficiency through its inherent feature selection and Bayesian backfitting MCMC algorithm [29], with fast prediction and robustness to missing data, outliers, and mixed data types [32]. Recent studies have leveraged BART to investigate health effects of multiple environmental exposures, highlighting its advantages in modeling complex interactions and cumulative risks from mixtures, whereas BKMR may face scalability issues [33–35].

In this study, we focus on tailoring the BART model for environmental mixture analysis to achieve both reliable identification of important chemicals and accurate prediction of health outcomes. Specifically, the modified BART accommodates both continuous and binary outcomes, allows for covariates adjustment, and incorporates biological grouping structures. Additionally, to facilitate interpretation of nonparametric response surface modeling results from both modified BART and BKMR, we introduce an lower-dimensional approximation technique based on the Generalized Additive Model (GAM) [36] to summarize the marginal effects of individual chemicals. We demonstrate the performance of the modified BART through extensive simulations and a case study using data from the National Health and Nutrition Examination Survey (NHANES) 2001–2002 cycle.

## Methods

We denote the sample size as N. For the $i^{th}$ (i=1,…, N) subject, we observe a health outcome $Y_{i}$, M environmental exposures ${𝐙}_{i}={\left(Z_{i\text{1}},Z_{i\text{2}},\cdots ,Z_{iM}\right)}^{⊤}$, and a set of covariates $X_{i}$. We first focus on a continuous outcome to describe the original BART model and our proposed modified BART model, followed by an extension to a binary outcome.

### Modified BART for environmental mixtures studies

The original BART model with a continuous outcome $Y_{i}$ can be defined as


$$Y_{i}=h\left({𝐙}_{i}\right)+\epsilon_{i},\epsilon_{i}\sim N\left(\text{0},\sigma^{\text{2}}\right).$$ (1)

The flexible surface function h(·) is defined with an ensemble of T trees as


$$h\left({𝐙}_{i}\right)=\sum_{t=\text{1}}^{T}g\left({𝐙}_{i};T_{t},M_{t}\right),$$

and each tree T is defined through g(.) as


$$g\left({𝐙}_{i};T_{t},M_{t}\right)=\sum_{\lambda \in \Lambda \left(T_{t}\right)}\overset{t}{\underset{\lambda }{\mu }}\phi_{\lambda }\left({𝐙}_{i};T_{t}\right).$$

**Fig 1:**
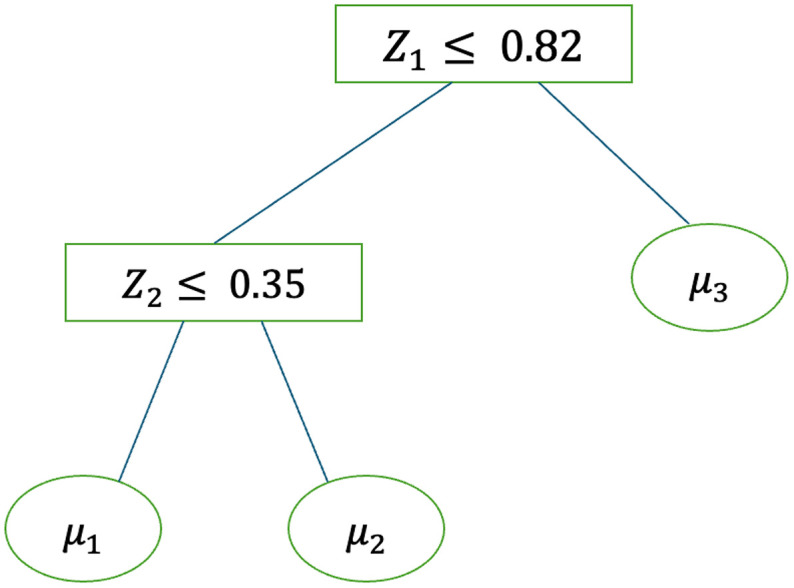
Schematic plot of an example binary decision tree𝐓 with exposures $\;(Z_{1},\;Z_{2})$. *Note*: The predicted values of the leaf nodes are $\begin{array}{c} \left\{\mu_{1},\;\mu_{2},\;\mu_{3}\right\} \end{array}$, with the branch splitting rules as $\begin{array}{c} \phi_{1}=I(Z_{2}\;\leq 0.35)*I(Z_{1}\leq 0.82) \end{array}$, $\begin{array}{c} \phi_{2}=I(Z_{2}>0.35)*I(Z_{1}\leq 0.82)\; \end{array}$, and $\begin{array}{c} \phi_{3}=I(Z_{1}>0.82)\; \end{array}$.

Here t (t=1,…, T) is the tree index, T is the total number of trees, $T_{t}$ denotes the $t^{th}$ decision tree topology structure, $\Lambda \left(T_{t}\right)$ denotes all leaf nodes associated with $T_{t}$, $\overset{t}{\underset{\lambda }{\mu }}$ represents the predicted values at leaf λ, $M_{t}=\left\{\overset{t}{\underset{\lambda }{\mu }},\;\lambda \in \Lambda \left(T_{t}\right)\right\}$ denotes all predicted values from the $t^{th}$ tree, and $\phi_{\lambda }(\cdot )$ defines the association of ${𝐙}_{i}$ to leaf node λ. Fig 1 illustrates a simple binary decision tree T with predictors $Z=\left(Z_{\text{1}},\;Z_{\text{2}}\right)$. The root node uses the splitting rule $Z_{\text{1}}\;\leq \text{0}.\text{8}\text{2}$. If this condition is not satisfied, it proceeds to the right, reaching a leaf node with predicted value $\mu_{\text{3}}$. Otherwise, it reaches an internal node with splitting rule $Z_{\text{2}}\;\leq \text{0}.\text{3}\text{5}$. If this holds, the path descends left to the leaf node with predicted value $\mu_{\text{1}}$; otherwise it terminates at the leaf node with predicted value $\mu_{\text{2}}$. Thus, we have $\phi_{\text{1}}(Z)=I(Z_{\text{2}}\;\leq \text{0}.\text{3}\text{5})*I(Z_{\text{1}}\leq \text{0}.\text{8}\text{2})$, $\phi_{\text{2}}(Z)=I(Z_{\text{2}}\;>\text{0}.\text{3}\text{5})*I(Z_{\text{1}}\leq \text{0}.\text{8}\text{2})$, $\phi_{\text{3}}(Z)=I(Z_{\text{1}}>\text{0}.\text{8}\text{2})$, and $g\left(Z;\text{T},\text{M}\right)=\mu_{\text{1}}\phi_{\text{1}}(Z)+\mu_{\text{2}}\phi_{\text{2}}(Z)+\mu_{\text{3}}\phi_{\text{3}}(Z)$ defines the binary decision tree, which corresponds to a step function. BART further sums these binary decision trees to create a more complicated step function, which can approximate a non-linear surface.

To further improve the smoothness of the fitted response surface, Linero and Yang [30] proposed soft BART by replacing the indicator function $\phi_{.}(z)$ with a soft decision rule, such as a logistic function $\psi_{.}(z)=\;\frac{\text{1}}{\text{1}+e^{-\frac{\left(z-c\right)}{\tau }}}$, where c is the cutpoint and τ is the bandwidth. Under this framework, within each tree, every subject is assigned to all leaves with probabilities determined by these logistic functions, and the estimate is an average of all the predicted values in these leaves weighted by the probabilities.

We make several modifications to soft BART to better suit environmental mixtures analysis, and name it the modified BART. Covariate adjustment is essential for epidemiological studies. Since these covariates are usually established risk factors, they need to remain in the model in fixed parametric forms rather than being combined with 𝐙 in the tree structure. Because the original soft BART model does not include a separate covariate term, we modify the model as


$$Y_{i}=\sum_{t=\text{1}}^{T}g\left({𝐙}_{i};T_{t},M_{t}\right)+\overset{⊤}{\underset{i}{𝐗}}\beta +\epsilon_{i},$$ (2)

where β is a vector of coefficients for the covariates ${𝐗}_{i}$, and ⊤ is the transpose. These covariates can be continuous or binary. Here we include covariate effects linearly, but more complicated non-linear forms can be incorporated if desired.

This model is fitted via a Bayesian backfitting MCMC algorithm, similar to BART and Soft BART. Briefly, in each MCMC iteration, we first computes $\overset{\prime }{\underset{i}{Y}}=Y_{i}-\sum_{t=\text{1}}^{T}g(Z_{i};T_{\text{t}},M_{\text{t}})$ using the current tree structures, and fit a Bayesian linear regression of $\overset{\prime }{\underset{i}{Y}}$ on $X_{i}$ to estimate β. Then the algorithm cycles through the T trees. For the t^th^ tree (t=1,…,T), it first computes the partial residuals leaving it out as $r_{it}=Y_{i}-\sum_{k\neq t}g\left(Z_{i};T_{\text{k}},M_{\text{k}}\right)-\overset{⊤}{\underset{i}{X}}\beta$, and then run a Metropolis-Hastings step to propose a modification $\overset{\prime }{\underset{\text{t}}{T}}$ (e.g., grow, prune, or change-split). The acceptance ratio is calculated based on the likelihood ratio of observing these partial residuals given $T_{\text{t}}$ and $\overset{\prime }{\underset{\text{t}}{T}}$, the ratio of observing $T_{\text{t}}$ and $\overset{\prime }{\underset{\text{t}}{T}}$ from the prior distribution, and the ratio of proposing $\overset{\prime }{\underset{\text{t}}{T}}{\text{ given}\;T}_{\text{t}}$ versus proposing $T_{\text{t}}$ given $\overset{\prime }{\underset{\text{t}}{T}}$. With soft BART, each subject is assigned to all the leaf nodes with logistic probabilities ψ(.); therefore, we integrate out the leaf parameters $M_{t}$ to compute a marginal likelihood used in the acceptance ratio calculation. If $\overset{\prime }{\underset{\text{t}}{T}}$ is accepted, the leaf parameters $M_{\text{t}}$ are updated using a Gaussian conjugate posterior; otherwise the parameters remain unchanged. The final step in each iteration updates other parameters, including the splitting probability for each exposure $p_{m}(m=\text{1},\ldots ,M)$ and variance parameters. This procedure is repeated many times to estimate model parameters, provide posterior inclusion probabilities (PIPs) for each exposure and predict outcomes.

Variable selection is accomplished through two mechanisms as in Linero and Yang [30]. First, we use a sparsity-inducing prior on the splitting probabilities at each exposure $p=(p_{\text{1}},p_{\text{2}},\ldots p_{M})$, and assume a Dirichlet prior $Dir(\frac{a}{M},\frac{a}{M},\;\ldots ,\frac{a}{M})$. When a is small, this prior encourages most splitting probabilities to be near zero and only a few exposures can be split. Second, we carefully choose the bandwidth parameter τ in the soft decision functions ψ(.), where a smaller τ further restrict the splitting and reduces its influence on the leaf weights. These two mechanisms jointly promote sparse trees to achieve variable selection.

The modified BART also allows incorporation of scientifically meaningful chemical grouping information. Specifically, we adopt the variable grouping prior discussed in Linero and Yang [30] to capture the hierarchical relationships inherent in grouped exposure data. When exposures Z are partitioned into G groups, with group g containing $N_{g}$ components, the inclusion probability for the $k^{th}\;$component in group g  is modeled as $p_{gk}=u_{g}\cdot \omega_{gk}$ ($k=\text{1},\;\text{2},\;\cdots ,N_{g},\;g=\text{1},\;\text{2},\;\cdots ,\;G),$ where $u_{g}$ is the group-level inclusion probability, and $\omega_{gk}$ is the within-group conditional inclusion probability. We assume Dirichlet priors for both u and $\omega_{g}$ to induce sparsity. This formulation yields variable selection at multiple levels, allowing identification of both important groups and important components within group.

For a binary outcome Y∈{0, 1}, we take a latent variable approach by assuming a continuous latent $Y^{*}$ modeled as in Equation (2) and letting $Y=I(Y^{*}>\text{0})$. In each MCMC iteration, given the current tree structures and parameters, we sample $Y^{*}$ from a truncated normal distribution conditional on Y,


$$\overset{*}{\underset{i}{Y}}|Y_{i}=\text{1}\;\sim \;N^{+}\left(\sum_{t=\text{1}}^{T}g\left({𝐙}_{i};T_{t},M_{t}\right)+\overset{\text{T}}{\underset{i}{X}}\beta ,\;\text{1}\right),$$

$$\overset{*}{\underset{i}{Y}}|Y_{i}=\text{0}\;\sim {\;N}^{-}\left(\sum_{t=\text{1}}^{T}g\left({𝐙}_{i};T_{t},M_{t}\right)+\overset{\text{T}}{\underset{i}{X}}\beta ,\;\text{1}\right),$$

where $N^{-}$ and $N^{+}$ are normal distributions truncated below and above 0, respectively. We then follow the same procedure above to update tree structures and parameters based on $\left(\overset{*}{\underset{i}{Y}},\;Z_{i},\;X_{i}\right),\;i=\text{1},\ldots ,\;N$. After fitting, the continuous prediction of $Y^{*}$ can be converted to Pr(Y=1) as $\Phi (\sum_{t=\text{1}}^{T}g\left({𝐙}_{i};T_{t},M_{t}\right)+\overset{\text{T}}{\underset{i}{X}}\beta )$, where Φ(.) is the standard normal cumulative distribution function.

More technical details on the modified BART are provided in S1 File, and the method is implemented in the R package *modBART* (https://github.com/Shiny1818/ModSoftBART_against_BKMR).

### Brief Overview of BKMR

In BKMR [17], $h\;={\left[h\left({𝐙}_{\text{1}}\right),\;h\left({𝐙}_{\text{2}}\right),\;\cdots ,\;h\left({𝐙}_{N}\right)\right]}^{⊤}$ in Equation (2) is modeled using a normal prior with mean **0** and a regularized Gaussian kernel K(·,·) defined as


$$K\left[h\left({𝐙}_{i}\right),h\left({𝐙}_{j}\right)\right]=\tau \cdot exp\left\{-\sum_{m=\text{1}}^{M}r_{m}{\left(Z_{im}-Z_{jm}\right)}^{\text{2}}\right\},$$

where i, j ∈{1, ⋯, N}, τ is a regularization parameter, and $r_{m}$ are auxiliary parameters that controls the smoothness of h(·). Inference is done through a Bayesian MCMC. Component-wise variable selection in BKMR is achieved by imposing a spike-and-slab prior on the parameters $r_{m}$ to identify important exposures within the mixture. When biological grouping information exists, BKMR can perform hierarchical variable selection with more technical details in Bobb et al.[17]. We used the R *bkmr* package (https://cran.r-project.org/web/packages/bkmr/index.html) to fit this model. For a binary outcome, a probit BKMR model [18] links a latent normal $Y^{*}$ to the observed outcome through $Y=I(Y^{*}>\text{0})$, similar to the modified BART model. When using the *kmbayes* function in the *bkmr* package to fit the probit BKMR, it is recommended to set est.h = TRUE to address issues with non-positive definite precision matrices, though this leads to much longer computational time.

### Marginal approximation to facilitate interpretation of chemical individual effect

Both modified BART and BKMR are flexible response surface models that can capture complicated non-linear relationships between exposures and outcomes, but their fitted surfaces can be difficult to interpret. BKMR provides useful visualization tools to inspect the effects of various exposure combinations at pre-selected quantiles [17], but it does not give a single overall marginal effect for each exposure.

To enhance interpretability for both modified BART and BKMR, we introduce a generalized additive model (GAM) based low-dimensional approximation technique proposed by Woody et al.[36]. Let $\hat{h}\left(Z_{\text{1}},\;Z_{\text{2}}\ldots \;,\;Z_{M}\right)$ denote the estimated response surface from either modified BART or BKMR. We approximate this multivariate surface by a sum of individual response curves,


$$\hat{h}\left(Z_{\text{1}},\;Z_{\text{2}},\;\ldots \;,\;Z_{M}\right)=\gamma_{\text{0}}+s_{\text{1}}\left({\text{Z}}_{\text{1}}\right)+s_{\text{2}}\left(Z_{\text{2}}\right)+\ldots +s_{M}\left(Z_{M}\right)+\;e,\;e\sim N\left(\text{0},\;\overset{\text{2}}{\underset{e}{\sigma }}\right).$$

where $s_{k}\left(Z_{k}\right),k=\text{1},\;\cdots ,\;M$ are smooth functions (e.g., splines, kernels, tensor products) of exposure $Z_{k}$. In this study, we used thin plate regression splines for their practical performance and theoretical properties [37]. As discussed in Woody et al.[36], this approximation can be restricted to a subset of important exposures identified by modified BART or BKMR. This approximation decomposes a complex response surface into additive one-dimensional response curves $s_{k}\left({\text{Z}}_{k}\right)$ to represent each exposure’s marginal effect averaged over the distribution of other exposures, an advantage over methods like partial dependence plots provided by BKMR that require fixing other exposures. Interactions between pre-specified exposures, such as $s\left(Z_{k},\;Z_{l}\right),\;k,l\in \left\{\text{1},\ldots ,\;M\right\}$, can also be included. Based on our experience, marginal effects and two-way interaction typically capture most variability for environmental mixtures data. We do not recommend higher order interactions because of interpretation difficulties and computational burden for GAMs. The effectiveness of this approximation is evaluated using an $R^{\text{2}}$ metric as outlined by Woody et al.[36], which measures how much variation in the posterior samples the approximation explains.

This approximation can be done through the *gam* function in R package *mgcv* (https://cran.r-project.org/web/packages/mgcv/index.html).

### Simulation Settings

We conducted extensive simulation studies to evaluate the performance of modified BART in settings reflecting environmental mixtures analyses, in comparison with BKMR. For all simulations, we used total sample size N=500, 1000 and 2000, equally divided into independent training and testing datasets (i.e., $N_{train}=N_{test}=\text{2}\text{5}\text{0},\;\text{5}\text{0}\text{0},\;\text{1}\text{0}\text{0}\text{0}$ respectively). Simulations were repeated 500 times.

We generated M=15 exposures from 𝐙~MVN(0,Σ), where Σ is a 15×15 covariance matrix structured as block-diagonal:


$$\Sigma =(\begin{array}{cccc} C_{\text{1}} & \text{0} & \text{0} & \text{0}\\ \text{0} & C_{\text{2}} & \text{0} & \text{0}\\ \text{0} & \text{0} & C_{\text{3}} & \text{0}\\ \text{0} & \text{0} & \text{0} & C_{\text{4}} \end{array}),$$

with $C_{\text{1}}$, $C_{\text{2}}$, $C_{\text{3}}$, and $C_{\text{4}}$ representing the covariance matrices for four exposure groups of dimensions 3×3, 4×4, 5×5, and 3×3, respectively. Off-diagonal elements were 0.6 in $C_{\text{1}}$, 0.3 in $C_{\text{2}}$, 0.75 in $C_{\text{3}}$, and 0.5 in $C_{\text{4}}$ to reflect moderate to high within group correlations. Exposures between groups were uncorrelated. We generated two covariates $𝐗=\left(X_{\text{1}},X_{\text{2}}\right),$ with $X_{\text{1}}\sim N(\text{0},\;\text{1})$ and $X_{\text{2}}\sim \text{Bernoulli}(\text{0}.\text{5})$, with coefficients $\beta_{\text{1}}=\text{0}.\text{5}$ and $\beta_{\text{2}}=\text{0}.\text{2}$ as in Equation (2).

We investigated three distinct h(.) functions to generate a continuous outcome Y,


$$h_{\text{1}}\left(𝐙\right)=f\left[\text{0}.\text{5}\left(Z_{\text{1}}+Z_{\text{2}}\right)+\text{0}.\text{3}\left(Z_{\text{4}}+Z_{\text{5}}+Z_{\text{6}}\right)\right],$$

$$h_{\text{2}}\left(𝐙\right)=\text{1}/\text{6}\left(Z_{\text{1}}+Z_{\text{2}}+\text{2}*Z_{\text{4}}+\text{2}*Z_{\text{5}}+\text{3}*Z_{\text{6}}+\text{1}/\text{2}*Z_{\text{1}}*Z_{\text{2}}+\text{1}/\text{3}*Z_{\text{4}}*Z_{\text{5}}*Z_{\text{6}}\right),$$

$$h_{\text{3}}\left(𝐙\right)=f\left[\text{1}/\text{6}\left(Z_{\text{1}}+Z_{\text{2}}+\text{2}*Z_{\text{4}}+\text{2}*Z_{\text{5}}+\text{3}*Z_{\text{6}}+\text{1}/\text{2}*Z_{\text{1}}*Z_{\text{2}}+\text{1}/\text{3}*Z_{\text{4}}*Z_{\text{5}}*Z_{\text{6}}\right)\right],$$

where $f(u)=\text{1}\text{0}*\left(\frac{\text{2}}{\text{1}+e^{-u}}-\text{0}.\text{3}*u-\text{1}\right)$ is a modified sigmoid function. This set of exposure-response functions capture non-linear main effects only ($h_{\text{1}}$), linear main effects with interactions ($h_{\text{2}}$), and non-linear main effects with interactions ($h_{\text{3}}$). The modified sigmoid function f(·) was chosen due to its smoothness and wide use to model does-response relationship in toxicology [38]. Out of the 15 exposures, only 5 exposures ($Z_{\text{1}},\;Z_{\text{2}}$ in group 1 and ${\;Z}_{\text{4}},Z_{\text{5}},Z_{\text{6}}$ in group 2) were assumed to be relevant to the outcome.

We fitted modified BART and BKMR on the training sets and obtained the predicted values $\hat{h}$ and $\hat{Y}$ on both training and testing sets. The modified BART model was trained with 20 or 50 trees, while we also tried 100 and 200 trees which showed negligible performance gains. To evaluate model predictive performance, we regressed $\hat{h}$ on the true h, separately for the training and testing sets, and reported the average intercept, slope, standard error (SE) of the regression model, and $R^{\text{2}}$ across replications. We also reported MSE for $\hat{Y}$ in both training and testing sets. Variable selection accuracy was assessed through PIPs for each exposure under component-wise variable selection, and through group and conditional PIPs under hierarchical variable selection. To visualize marginal effects, we applied the GAM approximation to the fitted exposure-response surfaces from both models. As a reference, we provided true individual dose-response curves from h(.) by fixing other exposures at their quartiles.

We separately implemented a recent fast BKMR algorithm which uses random Fourier features to accelerate the Gaussian process in the original BKMR algorithm [39]. Fast BKMR with 20 and 200 basis functions were compared with modified BART and BKMR under the same simulation setting with $N_{train}=N_{test}=\text{5}\text{0}\text{0}$. We also conducted a simulation with 50 exposures, with four groups of 15, 20, 10 and 5 exposures, to examine performance of modified BART in a higher dimensional setting with sparse signals. We kept the first two exposures in the first group and the first three within the second group to have effects on the outcome, while all the other simulations were kept the same. This introduced more irrelevant chemicals within each group. We reported the same metrics as previously discussed for these additional simulations.

For binary outcomes, we generated latent continuous variable $Y^{*}$ from $Y^{*}=h\left(Z\right)+X^{\text{T}}\beta +\epsilon$, and set $Y=I\left(Y^{*}>\text{0}\right)$, as discussed before. All other settings and fitting procedures matched the continuous case. To avoid the indefinite kernel issues in probit BKMR model, we set the est.h argument to *TRUE* in the *kmbayes* function, as suggested by the BKMR authors. In a separate simulation, we used the default setting est.h=FALSE to explore the failure rate of *kmbayes* for binary outcomes. To access model predictiveness, we regressed $\hat{h}$ on h and reported average intercept, slope, standard error and $R^{\text{2}}$ across simulation replications, and additionally reported AUC for this binary outcome setting. For variable selection, component-wise and hierarchical variable selection PIPs were reported. We also compared the GAM approximations for both models, with the true individual response curve from h(.) as references.

All experiments for BKMR and modified BART were conducted in the same computational environment on a high-performance computing cluster with Intel Xeon Gold 6252 and Platinum 8276 CPUs, using up to 80 nodes on 224 CPUs. We reported overall computational time, including model fitting and prediction sampling, averaged across replications.

### NHANES 2001–2002 data on the relationship between POPs and LTL

Gibson et al. [40] used data the 2001–2002 cycle National Health and Nutrition Examination Survey (NHANES) data to compare several mixtures analysis methods, including BKMR, for the relationship between leukocyte telomere length (LTL) and persistent organic pollutants (POPs). We used the same data and preprocessing procedure and restricted our analysis to individuals over the age of 20. We included 18 POPs with at least 60% of samples above the limit of detection. The 18 POPs exposures were categorized into three groups: non-dioxin-like polychlorinated biphenyls (non-dioxin-like PCBs), non-ortho PCBs, and toxic equivalent POPs (mPFD) [40,41]. The non-dioxin-like PCBs group included PCBs 74, 99, 138, 153, 170, 180, 187, and 194, the non-ortho PCBs group consisted of PCBs 126 and 169, and the mPFD group comprised of PCB 118, four dibenzo-furans (2,3,4,7,8-pncdf, 1,2,3,4,7,8-hxcdf, 1,2,3,6,7,8-hxcdf, 1,2,3,4,6,7,8-hxcdf), and three chlorinated dibenzo-p-dioxins (1,2,3,6,7,8-hxcdd, 1,2,3,4,6,7,8-hpcdd, 1,2,3,4,6,7,8,9-ocdd) [40]. Exposure values below the limits of detection (LOD) were imputed as $\text{LOD}/\sqrt{\text{2}}$. We adjusted for 13 covariates, including age, age^2^, sex (male, female), race/ethnicity (non-Hispanic black, non-Hispanic, white, Mexican American, other), educational attainment (college or more, some college, high school graduate, less than high school), BMI (≥30, 25–29.9, < 25), serum cotinine, white blood cell count, percent lymphocytes, percent monocytes, percent neutrophils, percent eosinophils, and percent basophils [40]. In the final dataset, all 18 exposures, LTL, and serum cotinine were log-transformed and scaled. Covariates related to blood cell counts and distributions were also scaled. After excluding observations with incomplete exposure and covariate information, the final dataset comprised 1003 participants. The study was approved by the Institutional Review Board of the National Center for Health Statistics.

We fitted both modified BART and BKMR, with and without grouping information, to compare findings. For the modified BART, we trained with 20 and 50 trees and performed both component-wise and hierarchical variable selection. For BKMR, we used a 100-point knot matrix constructed from the chemical data to ensure adequate coverage of the input space as recommended by Gibson et al.[40]. For both models, we summarized the variable selection results with component-wise, group-level and within-group PIPs. Marginal chemical effects from both models were approximated and visualized using GAM plots. We also provided partial marginal effects estimates from BKMR by fixing other exposures at their 1^st^, 2^nd^ and 3^rd^ quartiles for comparison.

## Results

### Simulation Results: Continuous Outcome

To assess prediction accuracy, we summarized the average intercept, slope, SE, and $R^{\text{2}}$ from regressing $\hat{h}$ on true h and MES for $\hat{Y}$, from modified BART and BKMR across 500 replications. Results for component-wise variable selection are summarized in S1 Table, and results for hierarchical variable selection are in Table 1. Optimal performance is indicated by an intercept close to zero, a slope close to one, high $R^{\text{2}}$, low SE, and low $MSE(\hat{Y}).$

**Table 1:** Simulation results for 15 exposures and a continuous outcome, with hierarchical variable selection for modified BART and BKMR.

	Training Dataset					Testing Dataset					Overall
	Int.	Slope	${𝐑}^{2}$	SE	$𝐌𝐒𝐄(\hat{Y})$	Int.	Slope	${𝐑}^{2}$	SE	$𝐌𝐒𝐄(\hat{Y})$	Computation Time
	$N_{train}$ = 250					$N_{test}$= 250					
$h_{\text{1}}(z)$											
modBART-20	−0.001	0.878	0.899	0.375	0.515	−0.002	0.847	0.821	0.504	0.813	2.00
modBART-50	0.000	0.893	0.904	0.369	0.468	−0.002	0.859	0.822	0.509	0.812	3.38
BKMR	0.000	0.645	0.679	0.568	0.925	−0.004	0.605	0.611	0.620	1.162	6.80
$h_{\text{2}}(z)$											
modBART-20	−0.001	0.958	0.959	0.185	0.432	0.000	0.945	0.954	0.194	0.552	1.86
modBART-50	−0.001	0.961	0.954	0.199	0.408	0.001	0.947	0.948	0.208	0.558	3.24
BKMR	0.015	0.660	0.679	0.432	0.734	0.016	0.642	0.652	0.445	0.832	6.84
$h_{\text{3}}(z)$											
modBART-20	0.002	0.911	0.922	0.317	0.478	0.004	0.884	0.861	0.422	0.716	2.03
modBART-50	0.002	0.920	0.923	0.317	0.445	0.004	0.892	0.860	0.427	0.718	3.51
BKMR	0.018	0.605	0.628	0.558	0.969	0.022	0.581	0.585	0.588	1.122	7.37
	$N_{train}$ = 500					$N_{test}$ = 500					
$h_{\text{1}}(z)$											
modBART-20	0.001	0.909	0.928	0.322	0.522	0.002	0.891	0.891	0.395	0.684	2.29
modBART-50	0.001	0.921	0.932	0.318	0.491	0.002	0.901	0.892	0.400	0.683	5.80
BKMR	−0.001	0.641	0.660	0.590	1.001	0.002	0.615	0.616	0.622	1.142	34.45
$h_{\text{2}}(z)$											
modBART-20	0.002	0.978	0.976	0.143	0.468	0.002	0.971	0.974	0.150	0.529	3.21
modBART-50	0.001	0.979	0.973	0.154	0.454	0.002	0.972	0.970	0.161	0.532	5.56
BKMR	0.017	0.667	0.677	0.439	0.774	0.018	0.659	0.661	0.449	0.814	50.49
$h_{\text{3}}(z)$											
modBART-20	0.002	0.933	0.943	0.276	0.498	0.005	0.918	0.906	0.352	0.643	1.93
modBART-50	0.002	0.940	0.943	0.275	0.477	0.005	0.924	0.905	0.355	0.644	3.13
BKMR	0.019	0.609	0.620	0.572	1.012	0.021	0.598	0.596	0.590	1.090	33.69
	$N_{train}\;$= 1000					$N_{test}$= 1000					
$h_{\text{1}}(z)$											
modBART-20	−0.002	0.939	0.961	0.241	0.512	−0.002	0.935	0.951	0.270	0.586	4.38
modBART-50	0.002	0.950	0.962	0.241	0.491	0.003	0.943	0.949	0.278	0.585	9.68
BKMR	−0.001	0.633	0.646	0.600	1.046	−0.001	0.618	0.621	0.619	1.133	276.77
$h_{\text{2}}(z)$											
modBART-20	−0.001	0.986	0.986	0.113	0.485	−0.001	0.983	0.984	0.117	0.518	4.45
modBART-50	−0.001	0.987	0.984	0.120	0.478	−0.001	0.983	0.982	0.125	0.520	9.10
BKMR	0.016	0.667	0.672	0.443	0.786	0.016	0.664	0.667	0.446	0.807	352.40
$h_{\text{3}}(z)$											
modBART-20	0.001	0.952	0.964	0.220	0.505	0.002	0.943	0.950	0.259	0.578	4.44
modBART-50	0.001	0.957	0.963	0.223	0.492	0.001	0.947	0.947	0.268	0.582	7.01
BKMR	0.020	0.609	0.616	0.577	1.038	0.020	0.602	0.604	0.586	1.081	333.17

*Note:* modBART-20 (50) denotes the modified BART model with number of trees set to 20 (50). Total sample size varied from 500, 1000 to 2000, with independently generated train and test datasets. True relationships between exposures and outcome varied from non-linear main effects only ($h_{\text{1}}$), linear main effects with interactions ($h_{\text{2}}$), to non-linear main effects with interactions ($h_{\text{3}}$). All simulations were replicated 500 times. We regressed estimated $\hat{h}$ on true h, and reported average intercept (Int.), slope, $R^{\text{2}}$, standard error (SE) for the regression, and MSE for $\hat{Y}$ ($MSE(\hat{Y})$). We also reported average overall computation time in minutes, including both model fitting and prediction sampling.

Under component-wise variable selection (S1 Table), BKMR performed slightly better than modified BART in nonlinear scenarios ($h_{\text{1}},\;h_{\text{3}}$) with a small sample size ($N_{train}=N_{test}=\text{2}\text{5}\text{0}$); however, the differences were minimal and became negligible as sample size increased. In linear settings ($h_{\text{2}}$), both methods showed comparable performance, with results close to optimal.

Under hierarchical variable selection (Table 1), modified BART performed consistently better than BKMR in both training and testing datasets, with intercepts closer to zero, slopes closer to one, higher $R^{\text{2}}$s, lower SEs and lower $MSE(\hat{Y})$. Notably, modified BART approached near-optimal performance as sample size increased, whereas BKMR’s performance improved little. For instance, when $N_{train}=N_{test}=\text{1}\text{0}\text{0}\text{0}$, in both linear and nonlinear scenarios, the estimated slope and $R^{\text{2}}$ values for BKMR on the test sets remained around 0.6−0.7, whereas modified BART achieved 0.93−0.95 for nonlinear settings and 0.98 for linear settings. In addition, $MSE(\hat{Y})$ was substantially lower with modified BART (approximately 0.50 and 0.59 for $h_{\text{1}}$ and $h_{\text{3}}$, 0.48 and 0.52 for $h_{\text{2}}$, in training and testing sets, respectively) than with BKMR (approximately 1.0 and 1.1 for $h_{\text{1}}$ and $h_{\text{3}}$, 0.79 and 0.81 for $h_{\text{2}}$, respectively).

Modified BART also demonstrated better scalability in all simulations, especially under hierarchical variable selection, in which it ran 15–80 times faster than BKMR for $N_{train}=N_{test}=\text{5}\text{0}\text{0}$ and 1000. For example, with $N_{train}=N_{test}=$1000, modified BART took about 4 minutes with 20 trees and under 10 minutes with 50 trees averaged across 500 replicates, while BKMR took 276–352 minutes across the three h(.) functions. Moreover, the number of trees (modBART-20 vs. modBART-50) in modified BART had minimal impact on results.

We also reported average PIPs to assess variable selection accuracy, for both the component-wise and hierarchical selections, with $h_{\text{1}}$ and $N_{train}=\text{2}\text{5}\text{0}$ across 500 replications in Table 2. Under component-wise variable selection, both methods accurately identifying important exposures, with similar PIPs (above 0.99 for all relevant exposures, and below 0.1 for all irrelevant exposures). Under hierarchical variable selection, both methods correctly assigned group PIPs near 1 to group 1 and 2, since $Z_{\text{1}},\;Z_{\text{2}}$ from group 1 and $Z_{\text{4}}-Z_{\text{6}}$ from group 2 affected the outcome. Larger differences were observed in conditional PIPs within the relevant groups. Modified BART assigned PIPs close to 1 for important exposures and below 0.28 for unimportant ones, while BKMR tended to distribute conditional probabilities more uniformly across important exposures (around 0.5 for group 1 and around 0.33 for group 2) and 0 for unimportant exposures. PIPs for $h_{\text{2}}$ and $h_{\text{3}}$ are provided in S4 and S5 Tables, respectively. Overall patterns were similar to $h_{\text{1}}$: both models successfully identified important exposures under component-wise variable selection and important groups under hierarchical variable selection. However, with component-wise variable selection, modified BART tended to assign slightly higher PIPs to some unimportant exposures. Under hierarchical variable selection, BKMR yielded conditional PIPs near zero or exactly zero for important exposures $Z_{\text{4}}$ and $Z_{\text{5}}$, selecting only $Z_{\text{6}}$ as important in group 2. This likely reflects differences in priors, with modified BART employs prior which allows equal chance of selection, whereas BKMR effectively favors only a single component from each selected group per iteration.

**Table 2:** Average PIPs for 15 exposures and a continuous outcome, with both component-wise and hierarchical variable selection for modified BART and BKMR. The true relationship is a non-linear main effect only model $h_{1}$, with $N_{train}=250$.

Exposures	Group	Relevance Indicator	Component-Wise Variable Selection		Hierarchical Variable Selection			
			Component PIP		Group PIP		Conditional PIP	
			BKMR	modBART-20	BKMR	modBART-20	BKMR	modBART-20
$N_{train}=\text{2}\text{5}\text{0}$								
$h_{\text{1}}(z)$								
Z_1_	1	1	1.000	0.999	1.000	1.000	0.516	0.999
Z_2_	1	1	1.000	0.999	1.000	1.000	0.484	0.999
Z_3_	1	0	0.085	0.096	1.000	1.000	0.000	0.241
Z_4_	2	1	1.000	0.993	1.000	1.000	0.346	0.998
Z_5_	2	1	1.000	0.993	1.000	1.000	0.349	0.998
Z_6_	2	1	1.000	0.992	1.000	1.000	0.305	0.997
Z_7_	2	0	0.064	0.083	1.000	1.000	0.000	0.272
Z_8_	3	0	0.039	0.066	0.192	0.093	0.203	0.225
Z_9_	3	0	0.039	0.069	0.192	0.093	0.200	0.218
Z_10_	3	0	0.040	0.069	0.192	0.093	0.201	0.226
Z_11_	3	0	0.043	0.067	0.192	0.093	0.206	0.235
Z_12_	3	0	0.034	0.068	0.192	0.093	0.190	0.240
Z_13_	4	0	0.041	0.070	0.175	0.088	0.339	0.368
Z_14_	4	0	0.044	0.073	0.175	0.088	0.330	0.352
Z_15_	4	0	0.046	0.076	0.175	0.088	0.331	0.380

*Note:* modBART-20 denotes the modified BART model with number of trees set to 20. All simulations were replicated 500 times. The relevance indicator denotes whether an exposure is relevant to the outcome. Component PIPs are derived from component-wise variable selection results, while group and conditional PIPs are derived from hierarchical variable selection.

**Fig 2:**
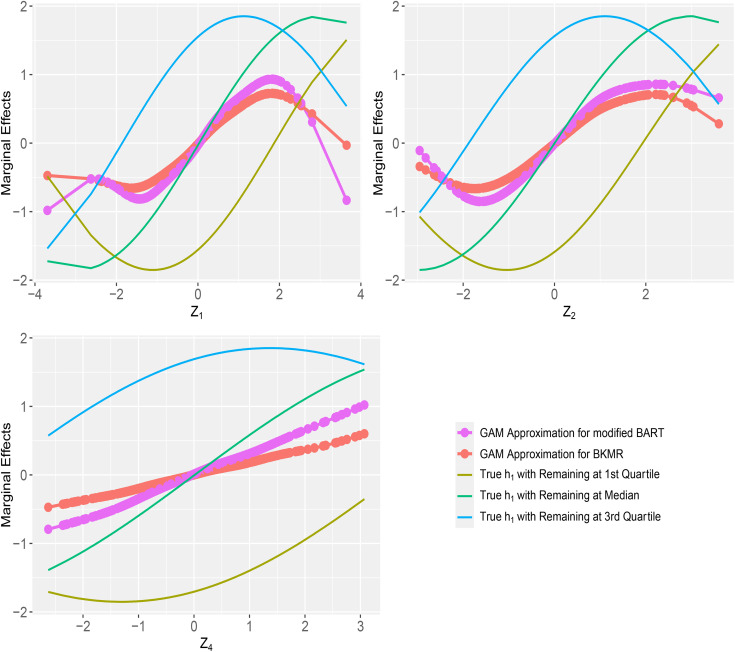
Average marginal effects for exposures ${𝐙}_{1}$, ${𝐙}_{2}$ and ${𝐙}_{4}$, in simulations with 15 exposures and a continuous outcome, using hierarchical variable selection for modified BART with 20 trees and BKMR. The true relationship is a non-linear main effects only model $\begin{array}{c} {\text{h}}_{1} \end{array}$, with $\begin{array}{c} {\text{\{N}}_{train}=N_{test}=250\} \end{array}$. *Note*: All simulations were replicated 500 times. The reference lines are true effects of each exposure by fixing all other exposures at their quartiles.

Average marginal effects of individual exposures through GAM approximation for $Z_{\text{1}},\;Z_{\text{2}}$ and $Z_{\text{4}}$ with $N_{train}=N_{test}=\text{2}\text{5}\text{0}$ are displayed in S1-1 ($h_{\text{1}}$), S1-2 ($h_{\text{2}}$) and S1-3 ($h_{\text{3}}$) Figures for component-wise variable selection, and in Fig 2 ($h_{\text{1}}$), S2-1 ($h_{\text{2}}$) and S2-2 ($h_{\text{3}}$) Figures for hierarchical variable selection. With component-wise variable selection, all marginal effects were approximated well with both methods, recovering the corresponding linear or non-linear relationships from the true model. For reference, we also overlaid the true marginal effects for these exposures by fixing the other exposures at their quartiles. The approximated marginal effects from both models generally followed the overall trends of the reference curves, which is consistent with interpretation as averages over these curves. In Fig 2, with the true relationship as a non-linear main effects only model $h_{\text{1}}$ and hierarchical variable selection, the marginal effects from modified BART and BKMR differed slightly, particularly in the tails, consistent with the differences in Table 1. However, both methods correctly captured the non-linear marginal effects for these exposures under $h_{\text{1}}$. However, consistent with the $h_{\text{2}}$ and $h_{\text{3}}$ hierarchical variable selection PIPs in S4 and S5 Tables, GAM plots showed that BKMR estimated near-zero marginal effects for $Z_{\text{4}}$ across the entire range under $h_{\text{2}}$ and $h_{\text{3}}$, while modified BART indicated clear effects (S2-1 and S2-2 Figs).

Comparison results for modified BART, BKMR and fast BKMR with $N_{train}=N_{test}=\text{5}\text{0}\text{0}$ under component-wise variable selection are shown in S8 Table. With 20 basis functions, computation time was reduced to a level comparable to modified BART, but prediction accuracy was substantially worse, with estimated slope < 0.5 and R^2^ < 0.6. Increasing the number of basis functions to 50, 100 and 200 increased computation time without improving prediction accuracy. Therefore, we did not pursue this approach further.

Hierarchical variable selection results with 50 exposures and $N_{train}=N_{test}=\text{5}\text{0}\text{0}$ are reported in S9 Table. Modified BART maintained superior prediction and variable selection accuracy, and computational scalability relative to BKMR.

### Simulation Results: Binary Outcome

Results for binary outcomes with both component-wise variable selection (S2 Table) and hierarchical variable selection (Table 3) showed patterns similar to those for continuous outcomes. Under component-wise variable selection, BKMR yielded slopes slightly closer to 1 and larger $R^{\text{2}}$ for nonlinear $h_{\text{1}}$ and $h_{\text{3}}$, while the results were similar with $h_{\text{2}}$. However, the computational advantages of modified BART were even more pronounced. For example, when $N_{train}=N_{test}=\text{5}\text{0}\text{0}$, run time was only about 2 minutes for modified BART with 20 trees, while BKMR required 106–136 minutes. When sample size increased to $N_{train}=N_{test}=\text{1}\text{0}\text{0}\text{0}$, BKMR became impractical with run time between 750 and 1020 minutes, while modified BART remained under 4 minutes. We also evaluated the default est.h=FALSE in *kmbayes* function. BKMR failed in more than 50% of replications at $N_{train}=N_{test}=\text{2}\text{5}\text{0}$ and in all replications at $N_{train}=N_{test}=\text{5}\text{0}\text{0}$ and  1000 (S3 Table).

**Table 3:** Simulation results for 15 exposures and a binary outcome, with hierarchical variable selection for modified probit BART and probit BKMR.

	Training Dataset					Testing Dataset					Overall
	Int.	Slope	${𝐑}^{2}$	SE	$𝐀𝐔𝐂(\hat{Y})$	Int.	Slope	${𝐑}^{2}$	SE	$𝐀𝐔𝐂(\hat{Y})$	Computation Time
	$N_{train}$ = 250					$N_{test}$= 250					
$h_{\text{1}}(z)$											
modBART-20	0.001	0.864	0.781	0.579	0.924	−0.003	0.834	0.732	0.645	0.873	1.05
modBART-50	0.000	0.859	0.783	0.572	0.926	−0.005	0.830	0.737	0.634	0.874	1.93
BKMR	0.000	0.593	0.617	0.599	0.874	−0.006	0.563	0.564	0.639	0.825	26.18
$h_{\text{2}}(z)$											
modBART-20	0.007	0.850	0.870	0.307	0.871	0.009	0.838	0.864	0.311	0.820	2.60
modBART-50	0.007	0.857	0.873	0.306	0.874	0.008	0.844	0.867	0.308	0.821	4.39
BKMR	0.018	0.546	0.619	0.409	0.821	0.019	0.529	0.594	0.416	0.775	46.86
$h_{\text{3}}(z)$											
modBART-20	0.020	0.921	0.824	0.506	0.917	0.022	0.899	0.790	0.554	0.870	1.06
modBART-50	0.017	0.923	0.827	0.501	0.919	0.020	0.902	0.795	0.548	0.870	1.84
BKMR	0.027	0.518	0.561	0.547	0.847	0.031	0.495	0.521	0.568	0.801	27.16
	$N_{train}$ = 500					$N_{test}$ = 500					
$h_{\text{1}}(z)$											
modBART-20	0.012	0.871	0.817	0.526	0.917	0.013	0.852	0.781	0.578	0.885	2.49
modBART-50	0.011	0.866	0.815	0.526	0.918	0.012	0.848	0.781	0.574	0.885	4.79
BKMR	0.008	0.546	0.605	0.565	0.853	0.007	0.531	0.575	0.586	0.827	187.28
$h_{\text{2}}(z)$											
modBART-20	−0.001	0.905	0.922	0.247	0.862	−0.002	0.901	0.920	0.250	0.831	2.16
modBART-50	−0.001	0.906	0.922	0.247	0.864	−0.002	0.901	0.920	0.250	0.831	4.21
BKMR	0.010	0.533	0.604	0.409	0.805	0.011	0.523	0.589	0.412	0.779	174.67
$h_{\text{3}}(z)$											
modBART-20	0.009	0.918	0.857	0.448	0.907	0.010	0.904	0.831	0.488	0.880	1.65
modBART-50	0.008	0.918	0.857	0.448	0.909	0.008	0.905	0.832	0.486	0.880	3.92
BKMR	0.018	0.469	0.535	0.518	0.825	0.022	0.454	0.507	0.531	0.800	149.07
	$N_{train}\;$= 1000					$N_{test}$= 1000					
$h_{\text{1}}(z)$											
modBART-20	0.006	0.895	0.857	0.467	0.914	0.006	0.884	0.831	0.508	0.893	4.37
modBART-50	0.005	0.898	0.857	0.468	0.916	0.005	0.888	0.832	0.508	0.892	5.94
BKMR	0.001	0.518	0.603	0.538	0.841	0.002	0.513	0.590	0.547	0.828	1343.21
$h_{\text{2}}(z)$											
modBART-20	0.004	0.930	0.952	0.197	0.853	0.004	0.929	0.951	0.198	0.836	3.43
modBART-50	0.004	0.928	0.951	0.200	0.854	0.004	0.927	0.950	0.201	0.836	7.25
BKMR	0.018	0.483	0.569	0.394	0.788	0.019	0.478	0.559	0.398	0.774	1313.09
$h_{\text{3}}(z)$											
modBART-20	0.005	0.924	0.885	0.398	0.902	0.006	0.914	0.865	0.433	0.885	2.98
modBART-50	0.005	0.923	0.884	0.400	0.903	0.005	0.913	0.865	0.431	0.884	6.14
BKMR	0.020	0.413	0.489	0.498	0.805	0.022	0.405	0.473	0.505	0.791	1377.38

*Note:* modBART-20 (50) denotes the modified probit BART model with number of trees set to 20 (50). BKMR denotes probit BKMR with est.h=TRUE. Total sample size varied from 500, 1000 to 2000, with independently generated train and test datasets. True relationships between exposures and outcome varied from non-linear main effects only ($h_{\text{1}}$), linear main effects with interactions ($h_{\text{2}}$), to non-linear main effects with interactions ($h_{\text{3}}$). All simulations were replicated 500 times. We regressed estimated $\hat{h}$ on true h, and reported average intercept (Int.), slope, $R^{\text{2}}$, standard error (SE) for the regression, and AUC for $\hat{Y}$ ($AUC(\hat{Y})$). We also reported average overall computation time in minutes, including both model fitting and prediction sampling.

With hierarchical variable selection, modified BART consistently outperformed BKMR across all settings, particularly at larger sample sizes. Slopes for modified BART were mostly around 0.9 in both training and testing datasets, while they were around 0.5 for BKMR. AUC values were also consistently higher for modified BART (0.82–0.93) than for BKMR (0.77–0.87). Computational time again was substantially shorter for modified BART and increase slowly with sample size. For example, when $N_{train}=N_{test}=\text{5}\text{0}\text{0}$, run time was under 3 minutes for modified BART with 20 trees, while BKMR required more than 150 minutes. With $N_{train}=N_{test}=\text{1}\text{0}\text{0}\text{0}$, BKMR required over 1300 minutes, whereas modified BART remained under 5 minutes.

PIPs for $h_{\text{1}}$ with $N_{train}=\text{2}\text{5}\text{0}$ (Table 4) further highlight the similarity of the two methods in identifying key exposures under component-wise variable selection and important chemical groups under hierarchical variable selection. However, modified BART showed better discrimination within groups, assigning conditional PIPs above 0.97 for all relevant exposures. Although it also assigned conditional PIP around 0.55 to two irrelevant exposures within group 1 and 2, likely due to the randomness induced by the prior, these were readily distinguishable from the truly important exposures with PIPs near 1. In contrast, BKMR tended to distribute importance across all relevant exposures, making it less straightforward to distinguish relevant from irrelevant exposures. PIPs for other settings are provided in the S6 and S7 Tables with similar patterns as the continuous settings.

**Table 4:** Average PIPs for 15 exposures and a binary outcome, with both component-wise and hierarchical variable selection for modified probit BART and probit BKMR. The true relationship is a non-linear main effect only model $h_{1}$, with $N_{train}=250$.

Exposures	Group	Relevance Indicator	Component-Wise Variable Selection		Hierarchical Variable Selection			
			Component PIP		Group PIP		Conditional PIP	
			BKMR	modBART-20	BKMR	modBART-20	BKMR	modBART-20
$N_{train}=\text{2}\text{5}\text{0}$								
$h_{\text{1}}(z)$								
Z_1_	1	1	1.000	0.999	1.000	1.000	0.799	0.998
Z_2_	1	1	1.000	0.999	1.000	1.000	0.201	0.999
Z_3_	1	0	0.351	0.440	1.000	1.000	0.000	0.537
Z_4_	2	1	0.996	0.974	1.000	1.000	0.550	0.985
Z_5_	2	1	0.997	0.981	1.000	1.000	0.228	0.989
Z_6_	2	1	0.991	0.964	1.000	1.000	0.222	0.978
Z_7_	2	0	0.311	0.412	1.000	1.000	0.000	0.573
Z_8_	3	0	0.267	0.394	0.343	0.351	0.199	0.302
Z_9_	3	0	0.250	0.388	0.343	0.351	0.188	0.302
Z_10_	3	0	0.262	0.394	0.343	0.351	0.208	0.307
Z_11_	3	0	0.261	0.392	0.343	0.351	0.203	0.309
Z_12_	3	0	0.252	0.394	0.343	0.351	0.202	0.311
Z_13_	4	0	0.247	0.382	0.334	0.350	0.334	0.440
Z_14_	4	0	0.249	0.381	0.334	0.350	0.328	0.439
Z_15_	4	0	0.249	0.386	0.334	0.350	0.337	0.440

*Note:* modBART-20 denotes the modified BART model with number of trees set to 20. All simulations were replicated 500 times. The relevance indicator denotes whether an exposure is relevant to the outcome. Component PIPs are derived from component-wise variable selection results, while group and conditional PIPs are derived from hierarchical variable selection.

**Fig 3:**
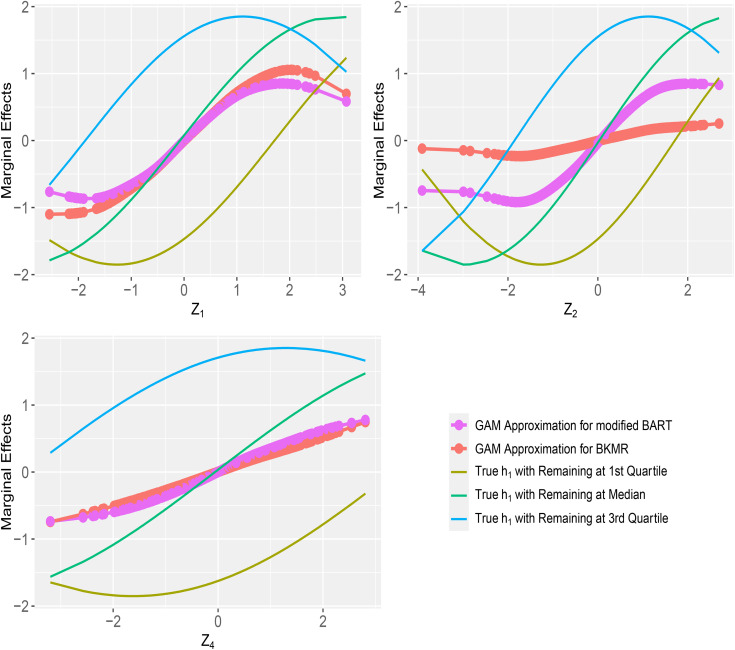
Average marginal effects for exposures ${𝐙}_{1}$, ${𝐙}_{2}$ and ${𝐙}_{4}$, in simulations with 15 exposures and a binary outcome, using hierarchical variable selection for modified probit BART with 20 trees and probit BKMR. The true relationship is a non-linear main effects only model $\begin{array}{c} {\text{h}}_{1} \end{array}$, with $\begin{array}{c} {\text{\{N}}_{train}=N_{test}=250\} \end{array}$. *Note*: All simulations were replicated 500 times. The reference lines are true effects of each exposure by fixing all other exposures at their quartiles.

Fig 3 shows GAM approximations of marginal effects for selected exposures $Z_{\text{1}},\;Z_{\text{2}}$ and $Z_{\text{4}}$ under $h_{\text{1}}$ with hierarchical variable selection. Both methods recovered the general shapes of the true partial effects similarly for $Z_{\text{1}}$ and $Z_{\text{4}}$, but modified BART more closely followed the nonlinear trends for $Z_{\text{2}}$ across quartiles, whereas BKMR smoothed over variation in these regions and produced flatter estimates. Results for other settings are provided in the Supplemental S3-1 to S3-3 for component-wise variable selection ($h_{\text{1}}$ to $h_{\text{3}}$) and S4-1 ($h_{\text{2}}$) and S4-2 ($h_{\text{3}}$) Figures for hierarchical variable selection. Consistent with findings above, marginal effects from both models were largely comparable under component-wise variable selection, aside from some divergence in the tails. Under hierarchical variable selection, however, more substantial differences emerged in both shape and magnitude of the marginal effects, with the modified BART more closely matching the reference curves than BKMR. This is most apparently in S4-1 Fig under the linear function $h_{\text{2}}$, where the marginal effect of $Z_{\text{4}}$ from modified BART was nearly parallel to the three reference lines as expected, while BKMR showed a smaller slope.

### NHANES data analysis results

We applied both modified BART and BKMR to the NHANES 2001–2002 data, to assess associations between 18 POPs and the outcome of interest, log-LTL. Results are summarized in Table 5.

**Table 5:** PIPs for 18 POPs and log-LTL from the NHANES 2001-2002 data, with both component-wise and hierarchical variable selection for modified BART and BKMR.

Chemicals	Group	Component-Wise Variable Selection		Hierarchical Variable Selection			
		Component PIP		Group PIP		Conditional PIP	
		BKMR	modBART-20	BKMR	modBART-20	BKMR	modBART-20
PCB74	non-dioxin-like PCBs	0.340	0.325	0.479	0.619	0.098	0.384
PCB99	non-dioxin-like PCBs	0.370	0.302	0.479	0.619	0.125	0.368
PCB138	non-dioxin-like PCBs	0.343	0.302	0.479	0.619	0.110	0.375
PCB153	non-dioxin-like PCBs	0.377	0.337	0.479	0.619	0.138	0.376
PCB170	non-dioxin-like PCBs	0.360	0.309	0.479	0.619	0.182	0.347
PCB180	non-dioxin-like PCBs	0.355	0.307	0.479	0.619	0.153	0.370
PCB187	non-dioxin-like PCBs	0.325	0.277	0.479	0.619	0.095	0.363
PCB194	non-dioxin-like PCBs	0.305	0.292	0.479	0.619	0.101	0.333
PCB118	mPFD	0.386	0.393	0.863	0.804	0.052	0.489
1,2,3,6,7,8-hxcdd	mPFD	0.321	0.287	0.863	0.804	0.011	0.335
1,2,3,4,6,7,8-hpcdd	mPFD	0.297	0.271	0.863	0.804	0.009	0.371
1,2,3,4,6,7,8,9-ocdd	mPFD	0.302	0.318	0.863	0.804	0.008	0.364
2,3,4,7,8-pncdf	mPFD	0.830	0.708	0.863	0.804	0.876	0.715
1,2,3,4,7,8-hxcdf	mPFD	0.311	0.315	0.863	0.804	0.013	0.346
1,2,3,6,7,8-hxcdf	mPFD	0.327	0.344	0.863	0.804	0.015	0.377
1,2,3,4,6,7,8-hxcdf	mPFD	0.349	0.360	0.863	0.804	0.017	0.398
PCB169	non-ortho PCBs	0.355	0.353	0.677	0.774	0.350	0.671
PCB126	non-ortho PCBs	0.463	0.398	0.677	0.774	0.650	0.837

*Note:* modBART-20 denotes the modified BART model with number of trees set to 20. Component PIPs are derived from component-wise variable selection results, while group and conditional PIPs are derived from hierarchical variable selection.

When grouping was ignored, both models general agreed across chemicals, assigning similar component PIPs. The most important chemical was 2,3,4,7,8-pncdf (PIPs = 0.708 for modified BART and 0.830 for BKMR), with PCB126 as the next most important (PIPs = 0.398 for modified BART and 0.463 for BKMR).

When grouping was incorporated, findings were consistent with Gibson et al.[40]. Group-level PIPs were broadly consistent across methods, with both identifying the mPFD group as the most important (group PIPs = 0.804 for modified BART and 0.863 for BKMR), followed by the non-ortho PCBs (group PIPs = 0.774 for modified BART and 0.677 for BKMR). Within the mPFD group, 2,3,4,7,8-pncdf was identified as the most important chemical (conditional PIPs = 0.715 for modified BART and 0.876 for BKMR). Both methods also identified PCB126 within the non-ortho PCBs group as an important exposure (conditional PIPs = 0.837 for modified BART and 0.650 for BKMR). Modified BART additionally assigned a higher conditional PIP = 0.671 to PCB169. Overall, the conditional PIP patterns were consistent with the simulation studies, where BKMR tended to favor the most important exposures within significant groups and modified BART produced more discriminative conditional PIPs for each exposure. Notably, conditional PIPs for PCB118 differed substantially (0.489 for modified BART vs. 0.052 for BKMR). As noted in Gibson et al. [40], PCB118 is a key mono-ortho dioxin-like PCBs whose toxicity differs from the other furans and dioxins in the mPFD group. In this analysis, we decided to retain the same grouping definition to align with Gibson et al. [40]. Despite this imperfect grouping, modified BART assigned PCB118 a much higher conditional PIP than BKMR, which may be desirable in practice.

**Fig 4:**
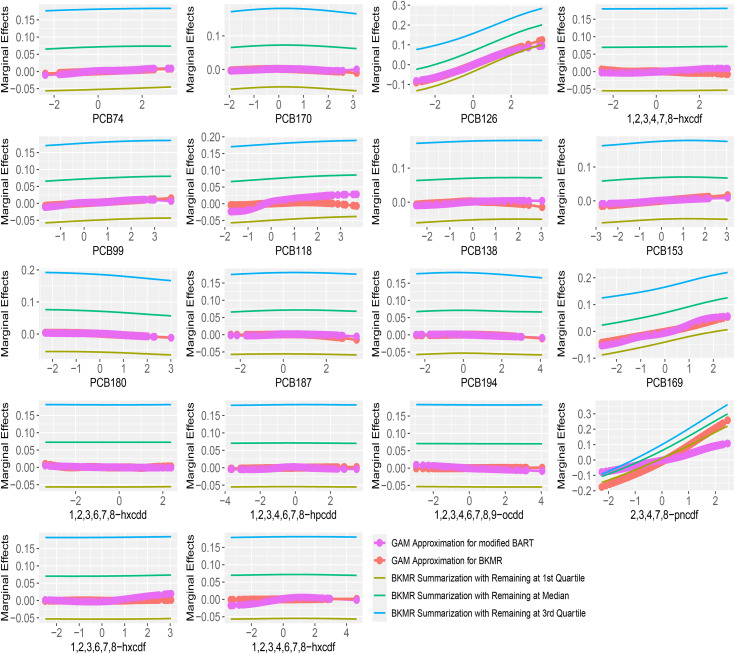
Marginal effects of 18 POPs on log-LTL from the NHANES 2001-2002 data, using hierarchical variable selection for modified BART with 20 trees and BKMR. *Note*: All chemicals were log-transformed and scaled. The reference lines are partial dependency curves from BKMR by fixing all other exposures at their quartiles.

Marginal effect estimates through GAM approximation with hierarchical variable selection are shown in Fig 4. The reference lines represent partial dependence curves derived from BKMR’s fitted results, without centering of the sampled posterior estimates. Most exposures showed approximately linear relationship, consistent with Gibson et al. [40]. For the most important exposure 2,3,4,7,8-pncdf, both modified BART and BKMR showed near-linear trajectories, although modified BART suggested a smaller effect size. For PCB 126 and PCB169, modified BART and BKMR produced similar marginal effect curves. Results from component-wise variable selection are provided in Supplementary S5 Fig. Most exposures showed similar curves, with minor tail divergences in some cases. For 2,3,4,7,8-pncdf, the overall patterns were consistent with the hierarchical variable selection results, but differences in marginal effect magnitudes were less pronounced.

## Conclusion and discussion

In this paper, we introduce and extend BART, a Bayesian tree ensemble method, to enhance the analytical toolkit for environmental chemical mixtures analysis. While the technical framework underlying our approach has been discussed in the statistical literature, our modified BART model allows for smooth exposure-response surfaces, adjustment for covariates, and hierarchical variable selection for grouped exposures. This extension enhances the capacity of BART to handle the complex structure of chemical mixtures, which is especially important in environmental epidemiology, where exposures often co-occur and interact in ways that challenge conventional models.

A major benefit of our approach is its ability to model group-level and component-level sparsity simultaneously. Unlike BKMR, which assumes equal probabilities for exposure inclusion within a group, our use of Dirichlet priors allows flexible group structures and improved differentiation of important mixture components. This is crucial for mixture studies, where identifying the “bad actor” within a mixture is essential for informing potential public health interventions. Our model structure avoids dilution of important signals and leads to clearer identification of key contributors to health outcomes. Across both continuous and binary outcomes, performance evaluations consistently demonstrated that modified BART outperformed BKMR in the context of hierarchical variable selection, and BKMR tended to underestimate conditional PIPs for exposures, potentially leading to unreliable conclusions. Moreover, the modified BART model offers notable advantages over BKMR in computational efficiency. By leveraging efficient C++ implementation, our model substantially reduces computation time, making it more practical for analyzing large-scale exposure data.

A further advantage of our approach is its robustness for binary outcomes, which are frequently encountered in environmental and public health research (e.g., presence or absence of disease). The default probit BKMR algorithm often fails with binary outcomes, especially with moderate and large sample sizes. Alternative strategies to avoid such failure can lead to degraded performance and substantially increased computation time. In comparison, our modified probit BART model maintains stable performance and reasonable computation time, highlighting its reliability in real-world mixtures applications.

Another contribution of our proposed framework is the integration of a low-dimensional approximation via GAM fitting. This technique improves the interpretability of accurate but complicated nonparametric response-surface models, including modified BART and BKMR, and makes it easier to understand marginal effects of individual exposures. This is particularly valuable in communicating results to policymakers and stakeholders in public health.

Although there are concerns regarding overfitting with machine learning methods, BART-based methods are specifically designed to mitigate overfitting and limit model complexity through strong Bayesian regularization. In particular, BART imposes priors that heavily penalize deep trees, ensuring that individual trees are shallow and contribute weakly to the overall fit. It also applies shrinkage to terminal node parameters to reduce overfitting within each tree. In addition, BART averages over a large number of trees, which reduces variance and stabilizes estimates. Together, these built-in mechanisms substantially reduce the risk of overfitting. We assessed predictive performance of modified BART with independent testing datasets in the simulations, and the results showed no evidence of overfitting.

Several algorithms have been proposed to mitigate the computational burden of the original BKMR, including fast BKMR [39], which uses a Fourier approximation to the kernels and Hamiltonian Monte Carlo for posterior sampling, and a variational inference algorithm [42]. We applied fast BKMR to our simulation settings with $N_{train}=N_{test}=\text{5}\text{0}\text{0}$ (S8 Table). With 20 basis functions, computation time was reduced to a level comparable to modified BART, but prediction accuracy was substaintially worse, with estimated slope < 0.5 and R^2^ < 0.6. Increasing the number of basis functions to 50, 100 and 200 increased computation time without improving prediction accuracy. Although fast BKMR is a promising approach for reducing computational burden, the accuracy of the Fourier approximation depends on several factors, including correlations among exposures, number of exposures, sample size and sparsity level. In addition, variable selection is not available in the current fast BKMR implementation. For the variation inference algorithm of BKMR, we were unable to obtain code and therefore did not include it in comparisons. We consider both approaches to have the potential for improving BKMR scalability, and would like to revisit comparisons when robust implementations become available with desirable features, such as variable selection and multiple outcome types.

For many environmental studies, it is important to adjust for confounders. However, we did not include confounder adjustment in the current manuscript, because BART focuses on variable selection and prediction and is not inherently a causal model. Although BART can be used to establish causal relationships, this requires working explicitly within a causal inference framework, which can be particularly complicated in the mixtures setting and is beyond the scope of this manuscript. It is not easy to establish a Directed Acyclic Graph (DAG) to provide a basis for confounder identification. Multiple exposures may influence the outcome through different biological pathways, and each pathway may be confounded by a distinct set of confounders. These confounder sets may overlap, but their effects can differ substantially across pathways. Whether confounders have overall effects on the outcome depends on which exposures and pathways are important, which cannot be decided prior to fitting BART. In additionally, including all potential confounders into BART could dramatically increase the number of input variables, resulting in unstable performance and heavy computational burden. Moreover, because exposures and potentially confounders would be modeled simultaneously, exposure effects could be incorrectly attributed to confounders, which is undesirable given that a primary goal of mixtures analysis is to identify toxic agents. Hahn et al. (2020) [43] also noted that regularized prediction models generally do not perform well when confounding is present, and instead proposed Bayesian Causal Forest (BCF), a BART-based causal inference method that allows clear separation of exposure effects and confounding effects. We are interested in extending such methods to mixtures analysis with explicit confounder adjustment.

In summary, we developed a modified BART model for estimating complex exposure-response relationships in environmental mixtures with covariate adjustment, improved handling of grouped exposures, greater computational efficiency, and robust performance across outcome types. Modified BART is a practical alternative to the BKMR, particularly with binary outcomes, hierarchical variable selection and larger sample sizes. To aid interpretation, we introduce a complementary post hoc approach using low-dimensional approximation via GAM fitting, which clarifies individual exposure effects without altering the core model. Future work will explore extending the model to survival outcomes and confounder adjustment to broaden its applicability in public health research.

## Supporting information

S1 File Details of modified BART. (DOCX)

S1 Table Simulation results for 15 exposures and a continuous outcome, with component-wise variable selection for modified BART and BKMR. (DOCX)

S2 Table Simulation results for 15 exposures and a binary outcome, with component-wise variable selection for modified probit BART and probit BKMR. (DOCX)

S3 TableSimulation results for 15 exposures and a binary outcome with component-wise variable selection for modified probit BART and default probit BKMR with est.h=FALSE.(DOCX)

S4 TableAverage PIPs for 15 exposures and a continuous outcome, with both component-wise and hierarchical variable selection for modified probit BART and probit BKMR.The true relationship is a linear main effects with interactions model $h_{\text{2}}$, with $N_{train}=\text{2}\text{5}\text{0}$.(DOCX)

S5 TableAverage PIPs for 15 exposures and a continuous outcome, with both component-wise and hierarchical variable selection for modified probit BART and probit BKMR.The true relationship is a non-linear main effects and interactions model $h_{\text{3}}$, with $N_{train}=\text{2}\text{5}\text{0}$.(DOCX)

S6 TableAverage PIPs for 15 exposures and a binary outcome, with both component-wise and hierarchical variable selection for modified probit BART and probit BKMR.The true relationship is a linear main effects and interactions model $h_{\text{2}}$, with $N_{train}=\text{2}\text{5}\text{0}$.(DOCX)

S7 TableAverage PIPs for 15 exposures and a binary outcome, with both component-wise and hierarchical variable selection for modified probit BART and probit BKMR.The true relationship is a non-linear main effects and interactions model $h_{\text{3}}$, with $N_{train}=\text{2}\text{5}\text{0}$.(DOCX)

S8 Table Simulation results for 15 exposures and a continuous outcome, with component-wise variable selection for modified BART, BKMR and fast BKMR. (DOCX)

S9 Table Simulation results for 50 exposures and a continuous outcome, with hierarchical variable selection for modified BART and BKMR. (DOCX)

S1-1 FigAverage marginal effects for exposures. $Z_{1}$, $Z_{2}$ and $Z_{4}$, in simulations with 15 exposures and a continuous outcome, using component-wise variable selection for modified BART with 20 trees and BKMR.The true relationship is a non-linear main effects only model $h_{\text{1}}$, with $N_{train}=N_{test}=\text{2}\text{5}\text{0}$.(DOCX)

S1-2 FigAverage marginal effects for exposures $Z_{1}$, $Z_{2}$ and $Z_{4}$, in simulations with 15 exposures and a continuous outcome, using component-wise variable selection for modified BART with 20 trees and BKMR.**The true relationship is a linear main effects and interactions model** $h_{\text{2}}$, **with** $N_{train}=N_{test}=\text{2}\text{5}\text{0}$.(DOCX)

S1-3 FigAverage marginal effects for exposures $Z_{1}$, $Z_{2}$ and $Z_{4}$, in simulations with 15 exposures and a continuous outcome, using component-wise variable selection for modified BART with 20 trees and BKMR.**The true relationship is a non-linear main effects and interactions model** $h_{\text{3}}$**, with.**(DOCX) $N_{train}=N_{test}=\text{2}\text{5}\text{0}.$

S2-1 FigAverage marginal effects for exposures $Z_{1}$, $Z_{2}$ and $Z_{4}$, in simulations with 15 exposures and a continuous outcome, using hierarchical variable selection for modified BART with 20 trees and BKMR.The true relationship is a linear main effects and interactions model $h_{\text{2}}$, with $N_{train}=N_{test}=\text{2}\text{5}\text{0}$.(DOCX)

S2-2 FigAverage marginal effects for exposures $Z_{1}$, $Z_{2}$ and $Z_{4}$, in simulations with 15 exposures and a continuous outcome, using hierarchical variable selection for modified BART with 20 trees and BKMR.The true relationship is a linear main effects and interactions model $h_{\text{2}}$, with $N_{train}=N_{test}=\text{2}\text{5}\text{0}$.(DOCX)

S3-1 FigAverage marginal effects for exposures $Z_{1}$, $Z_{2}$ and $Z_{4}$, in simulations with 15 exposures and a binary outcome, using component-wise variable selection for modified probit BART with 20 trees and probit BKMR.The true relationship is a non-linear main effects only model $h_{\text{1}}$, with $N_{train}=N_{test}=\text{2}\text{5}\text{0}$.(DOCX)

S3-2 FigAverage marginal effects for exposures $Z_{1}$, $Z_{2}$ and $Z_{4}$, in simulations with 15 exposures and a binary outcome, using component-wise variable selection for modified probit BART with 20 trees and probit BKMR.The true relationship is a linear main effects and interactions model $h_{\text{2}}$, with $N_{train}=N_{test}=\text{2}\text{5}\text{0}$.(DOCX)

S3-3 FigAverage marginal effects for exposures $Z_{1}$, $Z_{2}$ and $Z_{4}$, in simulations with 15 exposures and a binary outcome, using component-wise variable selection for modified probit BART with 20 trees and probit BKMR.The true relationship is a non-linear main effects and interactions model $h_{\text{3}}$, with $N_{train}=N_{test}=\text{2}\text{5}\text{0}$.(DOCX)

S4-1 FigAverage marginal effects for exposures $Z_{1}$, $Z_{2}$ and $Z_{4}$, in simulations with 15 exposures and a binary outcome, using hierarchical variable selection for modified probit BART with 20 trees and probit BKMR.The true relationship is a linear main effects and interactions only model $h_{\text{2}}$, with $N_{train}=N_{test}=\text{2}\text{5}\text{0}$.(DOCX)

S4-2 FigAverage marginal effects for exposures $Z_{1}$, $Z_{2}$ and $Z_{4}$, in simulations with 15 exposures and a binary outcome, using hierarchical variable selection for modified probit BART with 20 trees and probit BKMR.The true relationship is a non-linear main effects and interactions model $h_{\text{3}}$, with $N_{train}=N_{test}=\text{2}\text{5}\text{0}$.(DOCX)

S5 Fig Marginal effects of 18 POPs on log-LTL from the NHANES 2001–2002 data, using component-wise variable selection for modified BART with 20 trees and BKMR. (DOCX)
