# Supplementary material for: Tailoring Bayesian Additive Regression Trees (BART) for environmental mixture studies
Source: PLoS One. 2026 May 11;21(5):e0348002. doi: 10.1371/journal.pone.0348002 (PMC13160450; doi:10.1371/journal.pone.0348002)
Supplement: S1 File — (DOCX) [file pone.0348002.s001.docx]

**S1 File: Details of modified BART.**

In environmental mixtures study with soft BART, we represent the exposure-response function with a sum-of-tree ensemble,

$Y_{i}=h\left( \mathbf{Z}_{i} \right)+\mathbf{X}_{i}^{\top}\boldsymbol{\beta}+\epsilon_{i}$,

$h\left( \mathbf{Z}_{i} \right)=\sum_{t=1}^{T} g\left( \mathbf{Z}_{i};\mathcal{T}_{t},\mathcal{M}_{t} \right), \mathbf{Z}_{i}\boldsymbol{\in}\mathbb{R}^{M}$**,**

$g\left( \mathbf{Z}_{i};\mathcal{T}_{t},\mathcal{M}_{t} \right)=\sum_{\lambda\in\Lambda\left( \mathcal{T}_{t} \right)} \mu_{\lambda}^{t}\phi_{\lambda}\left( \mathbf{Z}_{i};\mathcal{T}_{t} \right)$,

$\phi_{\lambda}\left( \mathbf{Z}_{i};\mathcal{T}_{t} \right)= \prod_{b\in Anc(\lambda)} {\psi(\frac{Z_{ib}-c_{b}}{\tau_{t}})}^{1-R_{b}}\left[ 1-\psi(\frac{Z_{ib}-c_{b}}{\tau_{t}}) \right]^{R_{b}}$.

Where $\mathbf{Z}_{i}=\left( Z_{i1},Z_{i2},\cdots,Z_{iM} \right)^{\top}$ are environmental exposures, $\mathbf{X}_{i}$ are covariates, $\boldsymbol{\beta}$ is the vector of covariate coefficients, and $T$ stands for the total number of trees. $\mathcal{T}_{t}$ represents the $t^{th}$ tree’s topology structure and $\mathcal{M}_{t}$ is the cluster of leaf nodes associated with tree $t$. In the weight function $\phi_{\lambda}\left( \boldsymbol{\cdot} \right)$ for leaf node $\lambda$, $Anc(\lambda)$ is the set of ancestral nodes to $\lambda$, $Z_{ib}$ is the exposure selected at branch $b$ and $c_{b}$ is the cutting point. $\tau_{t}$ is the tree-specific bandwidth parameter and $\psi(\cdot)$ is a logistic gating function. $R_{b}=1$ if the path goes down right at branch $b$ and 0 otherwise. Compared to the original BART, the soft variant adopted both “soft” decision trees and sparsity-introducing Dirichlet prior on the splitting proportions. According to Linero and Yang(30), there’s a set of default priors specified for soft BART model and without further parameter tuning, we may expect a reasonable performance in most cases. In our exploration, we’ve compared different choices of tree numbers $T$ and arrived at 20 as the default level for environmental mixture study.

Before discussing the default priors of soft BART, a preprocessing routine on the dataset $(\boldsymbol{Z, Y})$ was recommended. In our implementation, the outcome $\boldsymbol{Y}$ was standardized and exposures $\boldsymbol{Z}$ were scaled to lie in the interval $\left[ 0,1 \right]$ through quantile transformation. As discussed in the literature(29-31), the transformation on $\boldsymbol{Y}$ is due to a data-informed prior specification strategy applied to leaf node parameters $\mu_{\lambda}^{t}\in\mathcal{M}_{t}$, which is assumed i.i.d. normally distributed. A linear transformation or standardization of $\boldsymbol{Y}$ can conveniently help us specify $\mu_{\lambda}^{t}\sim N(0, \frac{\sigma_{\mu}^{2}}{T})$. The preprocessing of $\boldsymbol{Z}$ works to ensure the prior invariant under monotone transformations, an appealing property of the original BART model(30).

*Priors Specification*

For component-wise variable selection, the Dirichlet prior for splitting proportions $\boldsymbol{p}=(p_{1}, \cdots, p_{M})$ is assumed to be $\boldsymbol{p}\sim Dir(a/M,\cdots, a/M)$, where $M$ indicates the total number of exposures. For the values of $a$, a default choice is $a/{(a+M)}\sim Beta(0.5, 1)$. The tree specific bandwidth parameter $\tau_{t}$ is assumed to independently sample from $Exp(0.1)$ to provide various choices of gating functions $\psi(\cdot)$. The error term’s standard deviation $\sigma$ has a half-Cauchy prior, $\sigma\sim{Cauchy}_{+}(0,\hat{\sigma})$, where $\hat{\sigma}$ is a lasso estimate of $\sigma$. For the tree structure, following Chipman et al.(29), we define a nonterminal node at depth $d$ with probability $\gamma{(1+d)}^{-\beta}$, and fix $\gamma=0.95$ and $\beta=2$, as no successes achieved when placing priors on those parameters(30). The last tree hyperparameter $\sigma_{\mu}$ also has half-Cauchy prior ${Cauchy}_{+}(0,0.25)$, where 0.25 is selected to make $\sigma_{\mu}$ have a median value at the default choices in the original BART model.

For hierarchical variable selection, we assume $M$ exposures are divided in to $G$ groups and the number of components included in group $g$ is $N_{g}$. We define the splitting proportions elements as $p_{gk}=u_{g}\cdot\omega_{gk} (g=1, 2, \cdots, G, k=1, 2, \cdots,N_{g})$ for the $k^{th}$ component in group $g$. Then following the variable grouping prior discussed in Linero and Yang(30), we assume

$u\mathcal{\sim D}\left( a/G,\cdots,a/G \right)$,

$\omega_{g}\mathcal{\sim D}\left( a_{c}/N_{g},\cdots,a_{c}/N_{g} \right)$,

where $a$ and $a_{c}$ serve as hyperparameters that control the overall sparsity of the model at the group and component levels, respectively. By default, these hyperparameters are set to 1, though they can be adjusted to reflect prior beliefs or data-driven requirements for stronger or weaker shrinkage. In the meanwhile, for a better adaptability and generalization, $a$ and $a_{c}$ are updated using scaled beta prime priors.

*Posterior Sampling*

As in Chipman et al.(29), we adopt the Bayesian backfitting MCMC algorithm to compute the tree ensemble estimates. For the incorporated linear covariates, we use a Bayesian linear model to obtain the posterior estimates on the coefficients.

At iteration $e$, we set $Y_{i}^{'}= Y_{i}-h\left( \mathbf{Z}_{i} \right)$ for $i=1, 2, \ldots, N$ and sample$\boldsymbol{\beta}$ using Bayesian linear model. After that, we set $Y_{i}^{*}= Y_{i}-\sum_{j\neq t} g(\mathbf{Z}_{i};\mathcal{T}_{j},\mathcal{M}_{j})-\mathbf{X}_{i}^{\top}\boldsymbol{\beta}$ for $i=1, 2, \ldots, N$, and sample $\mathcal{T}_{t}$ using Metropolis-Hastings proposal which consists of one of the moves set $\left\{ Birth, Death, Change \right\}$. $Birth$ makes a leaf node to a nonterminal node, $Death$ turns a nonterminal node into a leaf node, and $Change$ means changing the decision rule at a nonterminal node. Next, sampling $\mathcal{M}_{t}$ from a posterior normal distribution and $\tau_{t}$ using Metropolis-Hastings with the updated tree $\mathcal{T}_{t}$. The draws of $\mathcal{(T}_{t}, \mathcal{M}_{t}, \tau_{t})$ should loop over $t=1, 2, \ldots, T$.

After updating the tree parameters, we update $p$ from its posterior Dirichlet distribution under component-wise scenario, or update $u$ and $\omega_{g}(g=1, 2, \ldots, G)$ under hierarchical variable selection. The last step is sampling the variance parameter ($\sigma, \sigma_{\mu}$) and hyperparameters ($a, or (a, a_{c})$) appropriately. The technical details can be found in the supplementary materials by Linero and Yang(30). The iteration continues until reach the iteration limits and posterior samples are saved after burn-in period which can be used for estimation and uncertainty quantification.
