# Supplementary material for: Tailoring Bayesian Additive Regression Trees (BART) for environmental mixture studies
Source: PLoS One. 2026 May 11;21(5):e0348002. doi: 10.1371/journal.pone.0348002 (PMC13160450; doi:10.1371/journal.pone.0348002)
Supplement: S2 Table — (DOCX) [file pone.0348002.s003.docx]

S2 Table: Simulation results for 15 exposures and a binary outcome, with component-wise variable selection for modified probit BART and probit BKMR.

|  | Training Dataset | | | | | Testing Dataset | | | | | Overall |
| --- | --- | --- | --- | --- | --- | --- | --- | --- | --- | --- | --- |
|  | Int. | Slope | $R^{2}$ | SE | $AUC(\hat{Y})$ | Int. | Slope | $R^{2}$ | SE | $AUC(\hat{Y})$ | Computation Time |
|  | $N_{train}$ = 250 | | | | | $N_{test}$ = 250 | | | | |  |
| $h_{1}(z)$ | | | | | | | | | | | |
| modBART-20 | 0.000 | 0.863 | 0.774 | 0.591 | 0.927 | -0.004 | 0.830 | 0.723 | 0.657 | 0.871 | 0.89 |
| modBART-50 | 0.000 | 0.854 | 0.776 | 0.581 | 0.929 | -0.003 | 0.821 | 0.727 | 0.643 | 0.871 | 1.68 |
| BKMR | 0.003 | 1.214 | 0.834 | 0.695 | 0.959 | -0.003 | 1.165 | 0.807 | 0.728 | 0.883 | 23.41 |
| $h_{2}(z)$ | | | | | | | | | | | |
| modBART-20 | 0.006 | 0.855 | 0.862 | 0.320 | 0.878 | 0.007 | 0.842 | 0.856 | 0.321 | 0.819 | 1.56 |
| modBART-50 | 0.006 | 0.858 | 0.864 | 0.320 | 0.882 | 0.007 | 0.845 | 0.859 | 0.320 | 0.820 | 2.87 |
| BKMR | 0.000 | 0.919 | 0.855 | 0.360 | 0.887 | 0.001 | 0.898 | 0.850 | 0.354 | 0.819 | 29.02 |
| $h_{3}(z)$ | | | | | | | | | | | |
| modBART-20 | 0.019 | 0.926 | 0.822 | 0.513 | 0.921 | 0.022 | 0.902 | 0.787 | 0.561 | 0.869 | 1.09 |
| modBART-50 | 0.018 | 0.929 | 0.822 | 0.512 | 0.924 | 0.021 | 0.903 | 0.790 | 0.558 | 0.869 | 2.07 |
| BKMR | 0.004 | 1.130 | 0.856 | 0.559 | 0.942 | 0.008 | 1.099 | 0.833 | 0.589 | 0.875 | 25.16 |
|  | $N_{train}$ = 500 | | | | | $N_{test}$ = 500 | | | | |  |
| $h_{1}(z)$ | | | | | | | | | | | |
| modBART-20 | 0.012 | 0.864 | 0.811 | 0.531 | 0.918 | 0.013 | 0.844 | 0.774 | 0.583 | 0.884 | 1.42 |
| modBART-50 | 0.010 | 0.859 | 0.810 | 0.531 | 0.920 | 0.011 | 0.839 | 0.775 | 0.579 | 0.883 | 2.92 |
| BKMR | 0.015 | 1.061 | 0.896 | 0.459 | 0.937 | 0.015 | 1.045 | 0.882 | 0.485 | 0.897 | 105.60 |
| $h_{2}(z)$ | | | | | | | | | | | |
| modBART-20 | 0.008 | 0.903 | 0.919 | 0.253 | 0.864 | 0.008 | 0.897 | 0.915 | 0.256 | 0.831 | 2.31 |
| modBART-50 | 0.008 | 0.903 | 0.917 | 0.255 | 0.867 | 0.007 | 0.896 | 0.915 | 0.257 | 0.831 | 4.71 |
| BKMR | 0.004 | 0.920 | 0.924 | 0.247 | 0.863 | 0.004 | 0.915 | 0.921 | 0.249 | 0.833 | 135.99 |
| $h_{3}(z)$ | | | | | | | | | | | |
| modBART-20 | 0.012 | 0.910 | 0.852 | 0.454 | 0.909 | 0.016 | 0.897 | 0.828 | 0.490 | 0.878 | 2.07 |
| modBART-50 | 0.012 | 0.913 | 0.851 | 0.457 | 0.911 | 0.016 | 0.900 | 0.828 | 0.491 | 0.877 | 4.23 |
| BKMR | 0.003 | 1.025 | 0.902 | 0.404 | 0.921 | 0.005 | 1.014 | 0.889 | 0.428 | 0.885 | 128.20 |
|  | $N_{train}$= 1000 | | | | | $N_{test}$= 1000 | | | | |  |
| $h_{1}(z)$ | | | | | | | | | | | |
| modBART-20 | 0.007 | 0.890 | 0.854 | 0.469 | 0.914 | 0.007 | 0.880 | 0.828 | 0.511 | 0.893 | 2.91 |
| modBART-50 | 0.006 | 0.894 | 0.855 | 0.471 | 0.916 | 0.006 | 0.885 | 0.830 | 0.511 | 0.892 | 6.10 |
| BKMR | 0.005 | 1.024 | 0.937 | 0.337 | 0.927 | 0.004 | 1.020 | 0.932 | 0.350 | 0.903 | 749.23 |
| $h_{2}(z)$ | | | | | | | | | | | |
| modBART-20 | 0.006 | 0.927 | 0.950 | 0.201 | 0.854 | 0.007 | 0.925 | 0.949 | 0.202 | 0.835 | 3.12 |
| modBART-50 | 0.007 | 0.924 | 0.947 | 0.205 | 0.856 | 0.007 | 0.922 | 0.946 | 0.206 | 0.835 | 6.85 |
| BKMR | 0.004 | 0.930 | 0.952 | 0.196 | 0.853 | 0.004 | 0.928 | 0.951 | 0.197 | 0.836 | 1019.28 |
| $h_{3}(z)$ | | | | | | | | | | | |
| modBART-20 | 0.003 | 0.925 | 0.885 | 0.399 | 0.903 | 0.004 | 0.916 | 0.864 | 0.434 | 0.885 | 3.27 |
| modBART-50 | 0.002 | 0.925 | 0.884 | 0.401 | 0.905 | 0.003 | 0.916 | 0.865 | 0.434 | 0.884 | 7.06 |
| BKMR | -0.004 | 1.006 | 0.940 | 0.302 | 0.912 | -0.003 | 1.000 | 0.932 | 0.320 | 0.891 | 993.62 |

*Note:* modBART-20 (50) denotes the modified probit BART model with number of trees set to 20 (50). BKMR denotes probit BKMR with $est.h=TRUE$. Total sample size varied from 500, 1000 to 2000, with independently generated train and test datasets. True relationships between exposures and outcome varied from from non-linear main effects only ($h_{1}$), linear main effects with interactions ($h_{2}$), to non-linear main effects with interactions ($h_{3}$). All simulations were replicated 500 times. We regressed estimated $\hat{h}$ on true $h$, and reported average intercept (Int.), slope, $R^{2}$, standard error (SE) for the regression, and AUC for  $\hat{Y}$ ($AUC(\hat{Y})$). We also reported average overall computation time in minutes, including both model fitting and prediction sampling.
