# Supplementary material for: Tailoring Bayesian Additive Regression Trees (BART) for environmental mixture studies
Source: PLoS One. 2026 May 11;21(5):e0348002. doi: 10.1371/journal.pone.0348002 (PMC13160450; doi:10.1371/journal.pone.0348002)
Supplement: S3 Table — (DOCX) [file pone.0348002.s004.docx]

S3 Table: Simulation results for 15 exposures and a binary outcome with component-wise variable selection for modified probit BART and default probit BKMR with $est.h=FALSE$.

|  | Training Dataset | | | | Testing Dataset | | | | Overall |  |
| --- | --- | --- | --- | --- | --- | --- | --- | --- | --- | --- |
|  | Int. | Slope | $R^{2}$ | SE | Int. | Slope | $R^{2}$ | SE | Computational Time | # Failures |
|  | $N_{train}$ = 250 | | | | $N_{test}$ = 250 | | | |  |  |
| $h_{1}(z)$ |  |  |  |  |  |  |  |  |  |  |
| modBART-20 | 0.000 | 0.863 | 0.774 | 0.591 | -0.004 | 0.830 | 0.723 | 0.657 | 0.89 | 0 |
| modBART-50 | 0.000 | 0.854 | 0.776 | 0.581 | -0.003 | 0.821 | 0.727 | 0.643 | 1.68 | 0 |
| BKMR-default | -0.013 | 1.228 | 0.839 | 0.691 | -0.014 | 1.180 | 0.814 | 0.723 | 8.89 | 261 |
| $h_{2}(z)$ |  |  |  |  |  |  |  |  |  |  |
| modBART-20 | 0.006 | 0.855 | 0.862 | 0.320 | 0.007 | 0.842 | 0.856 | 0.321 | 1.56 | 0 |
| modBART-50 | 0.006 | 0.858 | 0.864 | 0.320 | 0.007 | 0.845 | 0.859 | 0.320 | 2.87 | 0 |
| BKMR-default | 0.003 | 0.932 | 0.866 | 0.342 | 0.006 | 0.915 | 0.859 | 0.345 | 14.64 | 377 |
| $h_{3}(z)$ |  |  |  |  |  |  |  |  |  |  |
| modBART-20 | 0.019 | 0.926 | 0.822 | 0.513 | 0.022 | 0.902 | 0.787 | 0.561 | 1.09 | 0 |
| modBART-50 | 0.018 | 0.929 | 0.822 | 0.512 | 0.021 | 0.903 | 0.790 | 0.558 | 2.07 | 0 |
| BKMR-default | 0.020 | 1.135 | 0.853 | 0.566 | 0.025 | 1.102 | 0.827 | 0.602 | 7.77 | 254 |
|  | $N_{train}$ = 500 | | | | $N_{test}$ = 500 | | | |  |  |
| $h_{1}(z)$ |  |  |  |  |  |  |  |  |  |  |
| modBART-20 | 0.012 | 0.864 | 0.811 | 0.531 | 0.013 | 0.844 | 0.774 | 0.583 | 1.42 | 0 |
| modBART-50 | 0.010 | 0.859 | 0.810 | 0.531 | 0.011 | 0.839 | 0.775 | 0.579 | 2.92 | 0 |
| BKMR-default | NA | NA | NA | NA | NA | NA | NA | NA | NA | 500 |
| $h_{2}(z)$ |  |  |  |  |  |  |  |  |  |  |
| modBART-20 | 0.008 | 0.903 | 0.919 | 0.253 | 0.008 | 0.897 | 0.915 | 0.256 | 2.31 | 0 |
| modBART-50 | 0.008 | 0.903 | 0.917 | 0.255 | 0.007 | 0.896 | 0.915 | 0.257 | 4.71 | 0 |
| BKMR-default | NA | NA | NA | NA | NA | NA | NA | NA | NA | 500 |
| $h_{3}(z)$ |  |  |  |  |  |  |  |  |  |  |
| modBART-20 | 0.012 | 0.910 | 0.852 | 0.454 | 0.016 | 0.897 | 0.828 | 0.490 | 2.07 | 0 |
| modBART-50 | 0.012 | 0.913 | 0.851 | 0.457 | 0.016 | 0.900 | 0.828 | 0.491 | 4.23 | 0 |
| BKMR-default | NA | NA | NA | NA | NA | NA | NA | NA | NA | 500 |
|  | $N_{train}$=1000 | | | | $N_{test}$ = 1000 | | | |  |  |
| $h_{1}(z)$ |  |  |  |  |  |  |  |  |  |  |
| modBART-20 | 0.007 | 0.890 | 0.854 | 0.469 | 0.007 | 0.880 | 0.828 | 0.511 | 2.91 | 0 |
| modBART-50 | 0.006 | 0.894 | 0.855 | 0.471 | 0.006 | 0.885 | 0.830 | 0.511 | 6.10 | 0 |
| BKMR-default | NA | NA | NA | NA | NA | NA | NA | NA | NA | 500 |
| $h_{2}(z)$ |  |  |  |  |  |  |  |  |  |  |
| modBART-20 | 0.006 | 0.927 | 0.950 | 0.201 | 0.007 | 0.925 | 0.949 | 0.202 | 3.12 | 0 |
| modBART-50 | 0.007 | 0.924 | 0.947 | 0.205 | 0.007 | 0.922 | 0.946 | 0.206 | 6.85 | 0 |
| BKMR-default | NA | NA | NA | NA | NA | NA | NA | NA | NA | 500 |
| $h_{3}(z)$ |  |  |  |  |  |  |  |  |  |  |
| modBART-20 | 0.003 | 0.925 | 0.885 | 0.399 | 0.004 | 0.916 | 0.864 | 0.434 | 3.27 | 0 |
| modBART-50 | 0.002 | 0.925 | 0.884 | 0.401 | 0.003 | 0.916 | 0.865 | 0.434 | 7.06 | 0 |
| BKMR-default | NA | NA | NA | NA | NA | NA | NA | NA | NA | 500 |

*Note:* modBART-20 (50) denotes the modified probit BART model with number of trees set to 20 (50). BKMR-default denotes probit BKMR with $est.h=FALSE.$ Total sample size varied from 500, 1000 to 2000, with independently generated train and test datasets. True relationships between exposures and outcome varied from non-linear main effects only ($h_{1}$), linear main effects with interactions ($h_{2}$), to non-linear main effects with interactions ($h_{3}$). All simulations were replicated 500 times. We regressed estimated $\hat{h}$ on true $h$, and reported average intercept (Int.), slope, $R^{2}$, and standard error (SE) for the regression. We also reported average overall computation time in minutes, including both model fitting and prediction sampling. We additionally reported how many simulations did not converge as $ failures.
