# Supplementary material for: Tailoring Bayesian Additive Regression Trees (BART) for environmental mixture studies
Source: PLoS One. 2026 May 11;21(5):e0348002. doi: 10.1371/journal.pone.0348002 (PMC13160450; doi:10.1371/journal.pone.0348002)
Supplement: S7 Table — The true relationship is a non-linear main effects and interactions model h3, with Ntrain=250. (DOCX) [file pone.0348002.s008.docx]

S7 Table: Average PIPs for 15 exposures and a binary outcome, with both component-wise and hierarchical variable selection for modified probit BART and probit BKMR. The true relationship is a non-linear main effects and interactions model $h_{3}$, with $N_{train}=250$.

| Exposures | Group | Relevance Indicator |  | Component-Wise  Variable Selection | |  | Hierarchical Variable Selection | | | |
| --- | --- | --- | --- | --- | --- | --- | --- | --- | --- | --- |
|  |  |  |  | Component PIP | |  | Group PIP | | Conditional PIP | |
|  |  |  |  | BKMR | modBART-20 |  | BKMR | modBART-20 | BKMR | modBART-20 |
| $N_{train}=250$ | | | | | | | | | | |
| $h_{3}(z)$ |  |  |  |  |  |  |  |  |  |  |
| Z_1_ | 1 | 1 |  | 0.817 | 0.800 |  | 0.984 | 0.985 | 0.489 | 0.779 |
| Z_2_ | 1 | 1 |  | 0.829 | 0.799 |  | 0.984 | 0.985 | 0.465 | 0.771 |
| Z_3_ | 1 | 0 |  | 0.338 | 0.460 |  | 0.984 | 0.985 | 0.046 | 0.424 |
| Z_4_ | 2 | 1 |  | 1.000 | 0.997 |  | 1.000 | 1.000 | 0.399 | 0.998 |
| Z_5_ | 2 | 1 |  | 1.000 | 0.998 |  | 1.000 | 1.000 | 0.044 | 0.999 |
| Z_6_ | 2 | 1 |  | 1.000 | 1.000 |  | 1.000 | 1.000 | 0.557 | 1.000 |
| Z_7_ | 2 | 0 |  | 0.292 | 0.414 |  | 1.000 | 1.000 | 0.000 | 0.637 |
| Z_8_ | 3 | 0 |  | 0.257 | 0.404 |  | 0.339 | 0.347 | 0.202 | 0.306 |
| Z_9_ | 3 | 0 |  | 0.251 | 0.399 |  | 0.339 | 0.347 | 0.210 | 0.316 |
| Z_10_ | 3 | 0 |  | 0.262 | 0.402 |  | 0.339 | 0.347 | 0.191 | 0.302 |
| Z_11_ | 3 | 0 |  | 0.268 | 0.408 |  | 0.339 | 0.347 | 0.199 | 0.317 |
| Z_12_ | 3 | 0 |  | 0.251 | 0.401 |  | 0.339 | 0.347 | 0.199 | 0.307 |
| Z_13_ | 4 | 0 |  | 0.250 | 0.393 |  | 0.355 | 0.350 | 0.326 | 0.441 |
| Z_14_ | 4 | 0 |  | 0.238 | 0.388 |  | 0.355 | 0.350 | 0.338 | 0.449 |
| Z_15_ | 4 | 0 |  | 0.260 | 0.396 |  | 0.355 | 0.350 | 0.336 | 0.448 |

*Note:* modBART-20 denotes the modified BART model with number of trees set to 20. All simulations were replicated 500 times. The relevance indicator denotes whether an exposure is relevant to the outcome. Component PIPs are derived from component-wise variable selection results, while group and conditional PIPs are derived from hierarchical variable selection.
