# Supplementary material for: Tailoring Bayesian Additive Regression Trees (BART) for environmental mixture studies
Source: PLoS One. 2026 May 11;21(5):e0348002. doi: 10.1371/journal.pone.0348002 (PMC13160450; doi:10.1371/journal.pone.0348002)
Supplement: S8 Table — (DOCX) [file pone.0348002.s009.docx]

S8 Table: Simulation results for 15 exposures and a continuous outcome, with component-wise variable selection for modified BART, BKMR and fast BKMR.

|  | Training Dataset | | | | Testing Dataset | | | | Overall | |
| --- | --- | --- | --- | --- | --- | --- | --- | --- | --- | --- |
|  | Int. | Slope | $R^{2}$ | SE | Int. | Slope | $R^{2}$ | SE | | Computational  Time |
|  | $N_{train}$ = 500 | | | | $N_{test}$ = 500 | | | | |  |
| $h_{1}(z)$ |  |  | |  |  |  | |  | |  |
| modBART-20 | -0.001 | 0.908 | 0.928 | 0.322 | 0.000 | 0.893 | 0.887 | 0.404 | | 3.35 |
| modBART-50 | -0.002 | 0.920 | 0.931 | 0.319 | -0.001 | 0.903 | 0.888 | 0.407 | | 6.76 |
| BKMR | -0.001 | 0.974 | 0.974 | 0.202 | -0.001 | 0.966 | 0.967 | 0.225 | | 41.35 |
| FastBKMR-20 | -0.001 | 0.435 | 0.541 | 0.442 | -0.001 | 0.363 | 0.403 | 0.487 | | 4.73 |
| FastBKMR-200 | -0.001 | 0.169 | 0.464 | 0.224 | 0.001 | 0.037 | 0.036 | 0.243 | | 46.27 |
| $h_{2}(z)$ |  |  |  |  |  |  |  |  | |  |
| modBART-20 | -0.001 | 0.977 | 0.976 | 0.145 | 0.000 | 0.972 | 0.973 | 0.152 | | 2.96 |
| modBART-50 | -0.001 | 0.979 | 0.972 | 0.156 | 0.000 | 0.973 | 0.969 | 0.164 | | 5.93 |
| BKMR | -0.001 | 0.977 | 0.977 | 0.140 | -0.001 | 0.971 | 0.974 | 0.149 | | 45.92 |
| FastBKMR-20 | -0.008 | 0.248 | 0.335 | 0.301 | -0.006 | 0.170 | 0.174 | 0.324 | | 4.62 |
| FastBKMR-200 | -0.004 | 0.137 | 0.380 | 0.164 | -0.002 | 0.022 | 0.019 | 0.164 | | 45.07 |
| $h_{3}(z)$ |  |  |  |  |  |  |  |  | |  |
| modBART-20 | 0.003 | 0.932 | 0.942 | 0.277 | 0.006 | 0.916 | 0.904 | 0.354 | | 3.22 |
| modBART-50 | 0.002 | 0.939 | 0.943 | 0.276 | 0.005 | 0.923 | 0.905 | 0.357 | | 5.72 |
| BKMR | -0.001 | 0.972 | 0.972 | 0.197 | 0.000 | 0.963 | 0.961 | 0.231 | | 41.43 |
| FastBKMR-20 | -0.006 | 0.425 | 0.530 | 0.427 | 0.000 | 0.353 | 0.391 | 0.468 | | 4.65 |
| FastBKMR-200 | -0.004 | 0.160 | 0.446 | 0.208 | -0.002 | 0.033 | 0.031 | 0.220 | | 45.17 |

*Note:* modBART-20 (50) denotes the modified BART model with number of trees set to 20 (50). FastBKMR-20 (200) denotes the fast BKMR with 20 (200) basis functions. Total sample size is 1000, with independently generated train and test datasets. True relationships between exposures and outcome varied from non-linear main effects only ($h_{1}$), linear main effects with interactions ($h_{2}$), to non-linear main effects with interactions ($h_{3}$). All simulations were replicated 500 times. We regressed estimated $\hat{h}$ on true $h$, and reported average intercept (Int.), slope, $R^{2}$, and standard error (SE) for the regression. We also reported average overall computation time in minutes, including both model fitting and prediction sampling.
