# Supplementary material for: Tailoring Bayesian Additive Regression Trees (BART) for environmental mixture studies
Source: PLoS One. 2026 May 11;21(5):e0348002. doi: 10.1371/journal.pone.0348002 (PMC13160450; doi:10.1371/journal.pone.0348002)
Supplement: S9 Table — (DOCX) [file pone.0348002.s010.docx]

S9 Table: Simulation results for 50 exposures and a continuous outcome, with hierarchical variable selection for modified BART and BKMR.

|  | Training Dataset | | | | | Testing Dataset | | | | | Overall |
| --- | --- | --- | --- | --- | --- | --- | --- | --- | --- | --- | --- |
|  | Intercept | Slope | $R^{2}$ | SE | $MSE(\hat{Y})$ | Intercept | Slope | $R^{2}$ | SE | $MSE(\hat{Y})$ | Computation Time |
|  | $N_{train}$ = 500 | | | | | $N_{test}$ = 500 | | | | |  |
| $h_{1}(z)$ |  |  |  |  |  |  |  |  |  |  |  |
| modBART-20 | -0.007 | 0.899 | 0.924 | 0.329 | 0.527 | -0.007 | 0.882 | 0.882 | 0.411 | 0.700 | 1.80 |
| modBART-50 | -0.007 | 0.914 | 0.929 | 0.322 | 0.492 | -0.008 | 0.895 | 0.885 | 0.409 | 0.694 | 3.39 |
| BKMR | -0.009 | 0.639 | 0.655 | 0.595 | 1.008 | -0.009 | 0.617 | 0.617 | 0.623 | 1.136 | 34.29 |
| $h_{2}(z)$ |  |  |  |  |  |  |  |  |  |  |  |
| modBART-20 | 0.001 | 0.969 | 0.972 | 0.153 | 0.450 | 0.002 | 0.962 | 0.970 | 0.160 | 0.531 | 1.56 |
| modBART-50 | 0.001 | 0.970 | 0.969 | 0.163 | 0.435 | 0.001 | 0.963 | 0.966 | 0.169 | 0.534 | 3.06 |
| BKMR | 0.014 | 0.664 | 0.675 | 0.437 | 0.762 | 0.015 | 0.657 | 0.662 | 0.446 | 0.811 | 32.35 |
| $h_{3}(z)$ |  |  |  |  |  |  |  |  |  |  |  |
| modBART-20 | -0.003 | 0.924 | 0.939 | 0.282 | 0.501 | -0.002 | 0.909 | 0.901 | 0.357 | 0.646 | 2.41 |
| modBART-50 | -0.003 | 0.933 | 0.941 | 0.280 | 0.479 | -0.002 | 0.916 | 0.903 | 0.358 | 0.644 | 4.51 |
| BKMR | 0.014 | 0.609 | 0.622 | 0.570 | 1.016 | 0.012 | 0.597 | 0.596 | 0.589 | 1.090 | 42.66 |

*Note:* modBART-20 (50) denotes the modified BART model with number of trees set to 20 (50). Total sample size is 1000, with independently generated train and test datasets. True relationships between exposures and outcome varied from non-linear main effects only ($h_{1}$), linear main effects with interactions ($h_{2}$), to non-linear main effects with interactions ($h_{3}$). All simulations were replicated 500 times. We regressed estimated $\hat{h}$ on true $h$, and reported average intercept (Int.), slope, $R^{2}$, standard error (SE) for the regression, and MSE for  $\hat{Y}$ ($MSE(\hat{Y})$). We also reported average overall computation time in minutes, including both model fitting and prediction sampling.
