# Supplementary material for: Tailoring Bayesian Additive Regression Trees (BART) for environmental mixture studies
Source: PLoS One. 2026 May 11;21(5):e0348002. doi: 10.1371/journal.pone.0348002 (PMC13160450; doi:10.1371/journal.pone.0348002)
Supplement: S1-3 Fig — The true relationship is a non-linear main effects and interactions model h3, with. (DOCX) Ntrain=Ntest=250. [file pone.0348002.s013.docx]

Figure S1-3: Average marginal effects for exposures $Z_{1}$, $Z_{2}$ and $Z_{4}$, in simulations with 15 exposures and a continuous outcome, using component-wise variable selection for modified BART with 20 trees and BKMR. The true relationship is a non-linear main effects and interactions model $h_{3}$, with $N_{train}=N_{test}=250.$****

*Note*: All simulations were replicated 500 times. The reference lines are true effects of each exposure by fixing all other exposures at their quartiles.
