# Supplementary material for: Tailoring Bayesian Additive Regression Trees (BART) for environmental mixture studies
Source: PLoS One. 2026 May 11;21(5):e0348002. doi: 10.1371/journal.pone.0348002 (PMC13160450; doi:10.1371/journal.pone.0348002)
Supplement: S4-1 Fig — The true relationship is a linear main effects and interactions only model h2, with Ntrain=Ntest=250. (DOCX) [file pone.0348002.s019.docx]

S4-1 Figure: Average marginal effects for exposures $Z_{1}$, $Z_{2}$ and $Z_{4}$, in simulations with 15 exposures and a binary outcome, using hierarchical variable selection for modified probit BART with 20 trees and probit BKMR. The true relationship is a linear main effects and interactions only model $h_{2}$, with $N_{train}=N_{test}=250$.

*Note*: All simulations were replicated 500 times. The reference lines are true effects of each exposure by fixing all other exposures at their quartiles.
