# Supplementary material for: Tailoring Bayesian Additive Regression Trees (BART) for environmental mixture studies
Source: PLoS One. 2026 May 11;21(5):e0348002. doi: 10.1371/journal.pone.0348002 (PMC13160450; doi:10.1371/journal.pone.0348002)
Supplement: S5 Fig — (DOCX) [file pone.0348002.s021.docx]

S5 Figure: Marginal effects of 18 POPs on log-LTL from the NHANES 2001-2002 data, using component-wise variable selection for modified BART with 20 trees and BKMR.

*Note*: All chemicals were log-transformed and scaled. The reference lines are partial dependency curves from BKMR by fixing all other exposures at their quartiles.
